# Identification of loci associated with conception rate in primiparous Holstein cows

**DOI:** 10.1186/s12864-019-6203-2

**Published:** 2019-11-12

**Authors:** Jennifer N. Kiser, Erin Clancey, Joao G. N. Moraes, Joseph Dalton, Gregory W. Burns, Thomas E. Spencer, Holly L. Neibergs

**Affiliations:** 1 Department of Animal Sciences and Center for Reproductive Biology, Washington State University, Pullman, WA United States; 2 Division of Animal Sciences, University of Missouri, Columbia, MO United States; 3 Department of Animal and Veterinary Science, University of Idaho, Caldwell, ID United States

**Keywords:** Cattle, Conception rate, Fertility, Genome-wide association analysis

## Abstract

**Background:**

Subfertility is a major issue facing the dairy industry as the average US Holstein cow conception rate (CCR) is approximately 35%. The genetics underlying the physiological processes responsible for CCR, the proportion of cows able to conceive and maintain a pregnancy at each breeding, are not well characterized. The objectives of this study were to identify loci, positional candidate genes, and transcription factor binding sites (TFBS) associated with CCR and determine if there was a genetic correlation between CCR and milk production in primiparous Holstein cows. Cows were bred via artificial insemination (AI) at either observed estrus or timed AI and pregnancy status was determined at day 35 post-insemination. Additive, dominant, and recessive efficient mixed model association expedited (EMMAX) models were used in two genome-wide association analyses (GWAA). One GWAA focused on CCR at first service (CCR1) comparing cows that conceived and maintained pregnancy to day 35 after the first AI (*n* = 494) to those that were open after the first AI (*n* = 538). The second GWAA investigated loci associated with the number of times bred (TBRD) required for conception in cows that either conceived after the first AI (*n* = 494) or repeated services (*n* = 472).

**Results:**

The CCR1 GWAA identified 123, 198, and 76 loci associated (*P* < 5 × 10^− 08^) in additive, dominant, and recessive models, respectively. The TBRD GWAA identified 66, 95, and 33 loci associated (*P* < 5 × 10^− 08^) in additive, dominant, and recessive models, respectively. Four of the top five loci were shared in CCR1 and TBRD for each GWAA model. Many of the associated loci harbored positional candidate genes and TFBS with putative functional relevance to fertility. Thirty-six of the loci were validated in previous GWAA studies across multiple breeds. None of the CCR1 or TBRD associated loci were associated with milk production, nor was their significance with phenotypic and genetic correlations to 305-day milk production.

**Conclusions:**

The identification and validation of loci, positional candidate genes, and TFBS associated with CCR1 and TBRD can be utilized to improve, and further characterize the processes involved in cattle fertility.

## Background

Subfertility remains a problem in the US dairy industry, impacting profitability and sustainability as poor fertility contributes to increased veterinary costs, culling rates, replacement rates, and additional inseminations to achieve a pregnancy [1, 2]. Within the dairy industry, there are several measures used to determine fertility in heifers and cows but conception rate (the number of cattle pregnant divided by the total number of cattle inseminated) is an important measure as it identifies the number of services required for a successful pregnancy to be reached. There has been a substantial decline in cow conception rates since the late 1950’s, with current Holstein cow conception rates near 35% [3–5]. This decline is likely due to numerous factors including changes in physiology, nutritional management of transition period and fresh cows, and selection of traits that might potentially have an adverse effect on fertility (e.g. production traits) [3, 4, 6, 7]. As milk production hinges on successful pregnancies, any antagonistic relationship between fertility and production traits is problematic. Conflicting reports exist, however, as to the exact nature of the relationship between fertility and reproduction [8, 9].

After years focusing on the incorporation of management practices to improve fertility, the dairy industry has recently turned to genomic selection to further enhance fertility. Genomic selection has been widely used in the dairy industry since the introduction of genomic evaluations in 2009 with over a million cattle having been genotyped [10]. Currently, most calves are tested within a month of age to allow producers to make selection decisions earlier, reducing costs of raising calves that will not be kept as replacements. Studies have shown the positive impact genomic selection has had on the dairy industry, with García-Ruiz et al. [11] reporting that genetic improvement for lowly heritability traits in US Holsteins has improved by a staggering 300–400% within a 7 year time period. Similarly, the inclusion of heifer and cow conception rates traits into selection indices has proven to be successful even though the genetic basis of subfertility in dairy cows is poorly characterized [12, 13]. One way to better understand the genetic basis of subfertility is to identify loci associated with cow conception rate through a genome-wide association analysis (GWAA). Once identified, these loci may be used with genomic selection to improve fertility and to provide insight into how specific loci elicit physiological effects that lead to pregnancy (and pregnancy loss) in cows and their effect on milk production in lactating cows. Therefore, the objectives of this study were to identify loci, positional candidate genes and transcription factor binding sites (TFBS) associated with cow conception rate (CCR) at first service (CCR1) and after repeated services (TBRD) in primiparous US Holsteins, and determine if there was evidence of a genetic correlation with loci associated with CCR1, TBRD, and milk production.

## Results

### Genome-wide association analyses

**Fig. 1:**
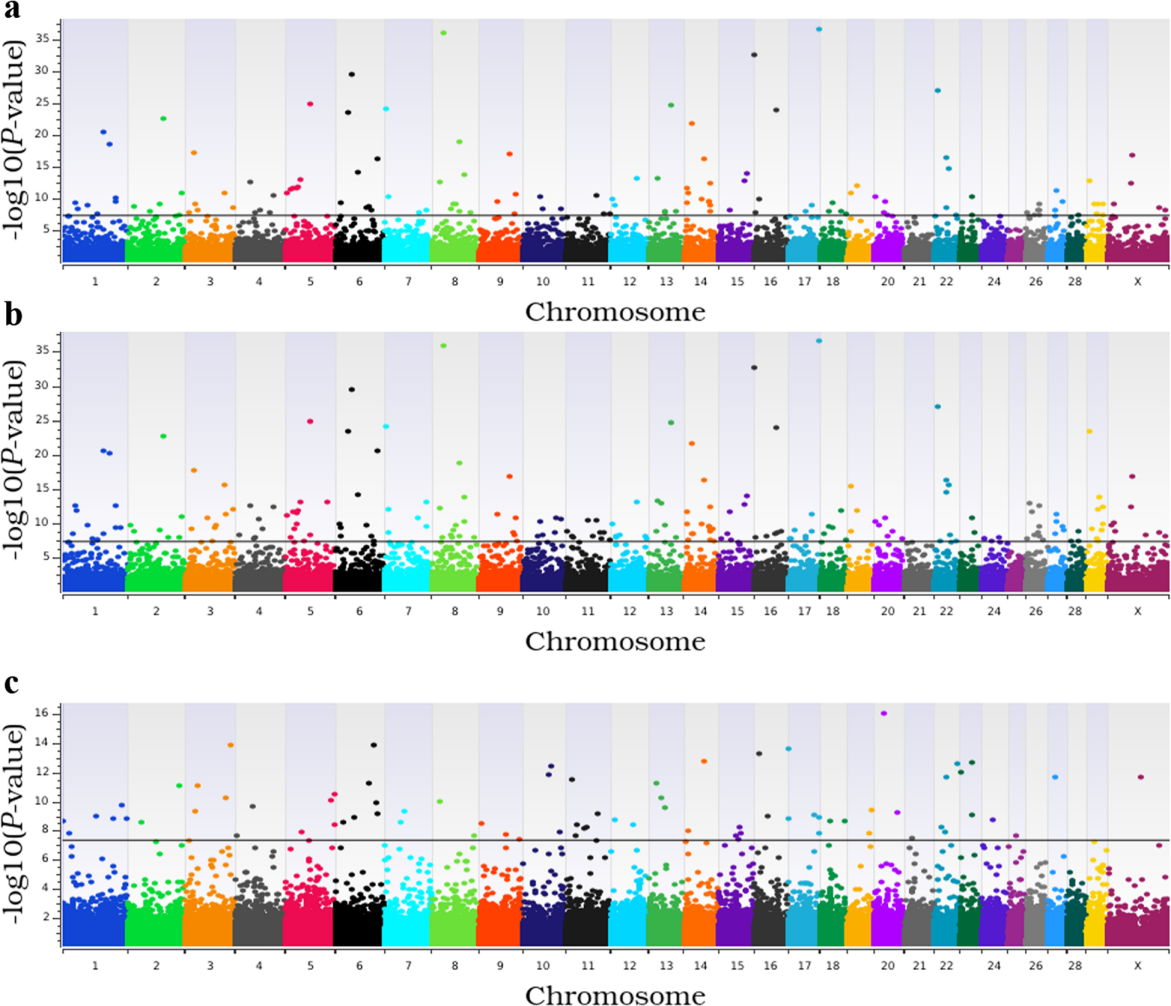
Manhattan plots for genome-wide association analyses for conception rate at first breeding. Panels **a**, **b**, and **c** present results from the additive, dominant, and recessive models, respectively. Single nucleotide polymorphisms are represented by a single dot. Bovine chromosomes are listed on the x-axis. Negative log10 (*P* values) ≥ 7.3 (black line) on the y-axis provided evidence for association (*P* < 5.0 × 10^− 08^)

**Fig. 2:**
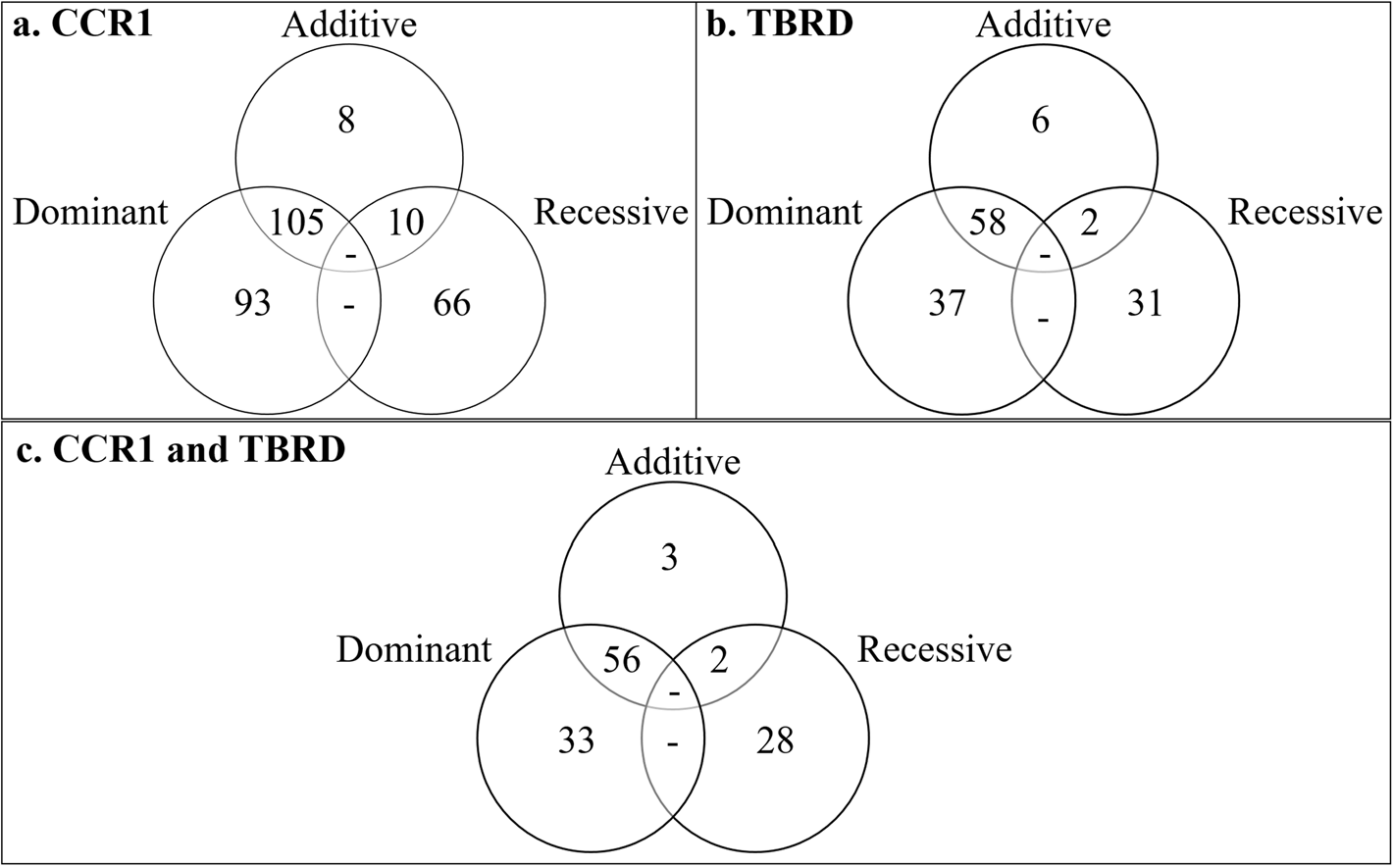
Relationships of loci identified between genotypic models and phenotypes. Panel **a** compares loci associated with conception rate at first breeding (CCR1) across the three genotypic models. Panel **b** compares loci associated with number of times bred to conception (TBRD) across genotypic models. Panel **c** compares loci across both phenotypes and all genotypic models

There were 123 (Fig. 1a), 198 (Fig. 1b) and 76 (Fig. 1c) loci that were associated with CCR1 in the additive, dominant, and recessive models, respectively (Fig. 2a; see Additional file 2: Table S1). The estimated heritability of CCR1 was 0.58 ± 0.06. The five most significant loci associated with CCR1 in the additive and dominant models were shared and contained six positional candidate genes, while the five most significant loci in the recessive model contained ten positional candidate genes and two TFBS (Table 1). An additional 263 positional candidate genes were identified in the remaining loci associated with CCR1.

**Table 1:** Top loci associated with conception rate to first breeding and number of breedings to conception

BTA^1^	BP Position^2^	SNP ID^3^	Model^4^	CCR1 *P*-value^5^	TBRD *P*-value^6^	Positional Candidate Gene(s)^7^	TFBS^8^
3	111,540,905 (110,948,044)	*rs133945887*	Recessive	1.33 × 10^−14^	7.25 × 10^−12^	*GJB5, GJB4*	*–*
6	38,447,022 (37,013,919)	*rs109381958*	Additive Dominant	4.31 × 10^−30^ 4.31 × 10^− 30^	4.39 × 10^− 26^ 4.39 × 10^− 26^	*–*	*–*
6	93,660,111 (91,908,296)	*rs132854961*	Recessive	1.56 × 10^− 14^	8.38 × 10^− 12^	***CCNI****, LOC101901983*	***–***
8	29,615,889 **(**29,565,411)	*rs134405734*	Additive Dominant	1.42 × 10^− 36^ 1.42 × 10^− 36^	1.25 × 10^− 24^ 1.25 × 10^− 24^	*ZDHHC21*	*–*
16	387,363 (591,660)	*rs133778157*	Additive Dominant	2.99 × 10^− 33^ 2.99 × 10^− 33^	5.68 × 10^− 28^ 5.68 × 10^− 28^	*LOC787642, LOC787620, LOC787667*	*–*
16	10,340,087 (9,739,230)	*rs137501137*	Recessive	5.27 × 10^− 14^	1.12 × 10^− 13^	*–*	*NF-1, Cutl1*
17	74,944,074 (72,930,491)	*rs133921184*	Additive Dominant	3.35 × 10^−37^ 2.93 × 10^− 37^	2.59 × 10^− 23^ 5.98 × 10^− 22^	*ARVCF, COMT*	*–*
20	23,886,196 (23,863,171)	*rs41663374*	Recessive	9.41 × 10^− 17^	1.58 × 10^− 11^	***DHX29****, LOC107131567*	*–*

^1^Chromosome location of the locus

^2^Single nucleotide polymorphism (SNP) location as measured by numbered nucleotides in reference to the UMD 3.1 genome assembly (http://bovinegenome.org/?q=node/61; accessed 15 September 2016) or the ARS 1.2 genome assembly (https://www.animalgenome.org/repository/cattle/UMC_bovine_coordinates/; accessed 19 September 2018) in parentheses

^3^The most significant SNP in the locus associated cow conception rate as identified by *rs* number which is a reference number assigned to markers submitted to the National Center for Biotechnology Information SNP database (https://www.ncbi.nlm.nih.gov/projects/SNP/; accessed 2 April 2018)

^4^Genome-wide association model

^5^Significance (*P-*value) of the most significant SNP associated with cow conception rate at first service (CCR1)

^6^Significance (*P-*value) of the most significant SNP associated with number of services per conception (TBRD)

^7^Positional candidate genes are defined as genes that are located within 17.8 kb on either side of the associated SNP(s)

^8^Transcription factor binding sites (TFBS) as identified using the Alggen Promo database (http://alggen.lsi.upc.es/cgi-bin/promo_v3/promo/promoinit.cgi?dirDB=TF_8.3; accessed 26 July 2018)

**Fig. 3:**
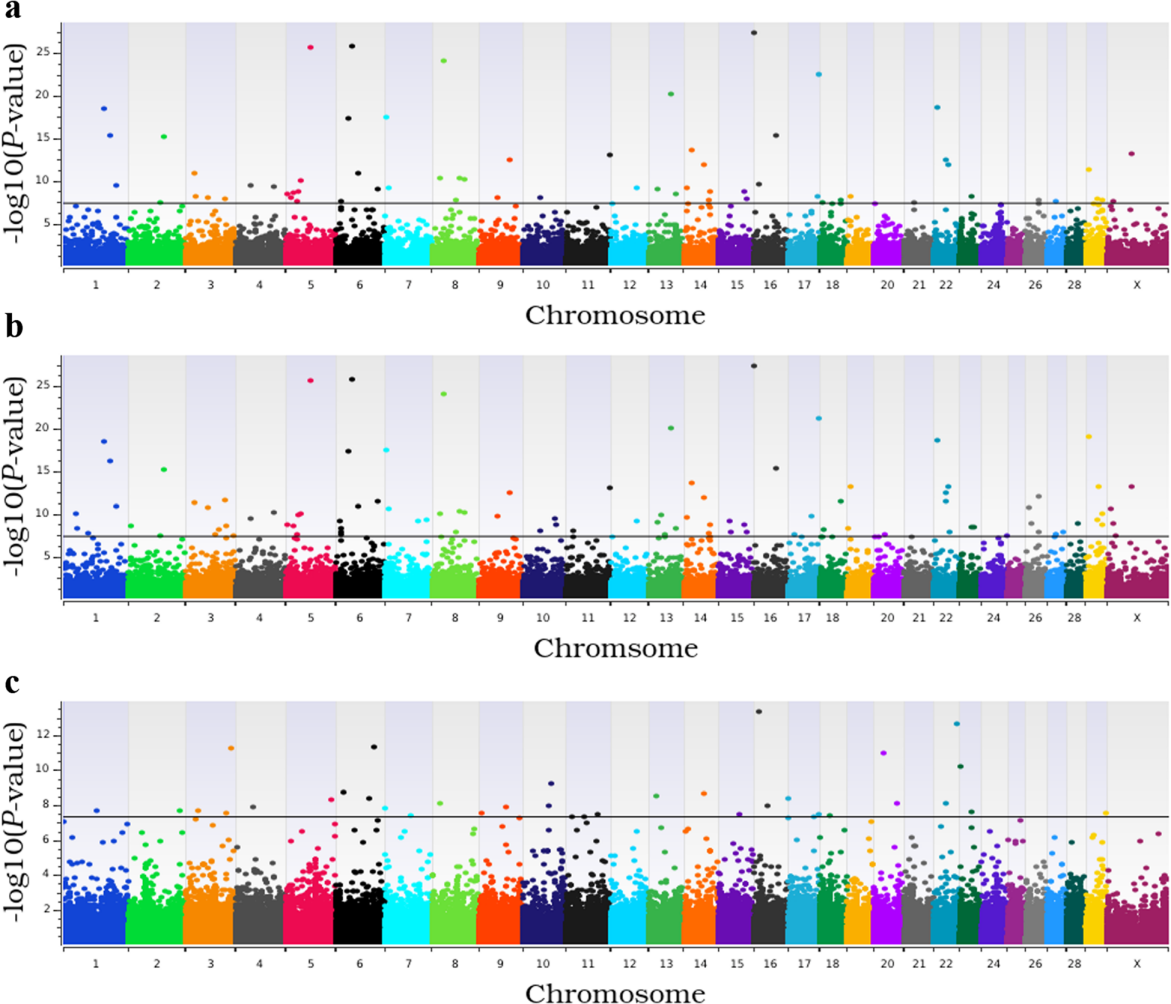
Manhattan plots for genome-wide association analyses for number of times bred to conception. Panels **a**, **b**, and **c** present results from the additive, dominant, and recessive models respectively. Single nucleotide polymorphisms are represented by a single dot. Bovine chromosomes are listed on the x-axis. Negative log10 (*P* values) ≥ 7.3 (black line) on the y-axis provided evidence for association (*P* < 5.0 × 10^− 08^)

For TBRD, 66 loci were associated in the additive model (Fig. 3a), 95 loci were associated in the dominant model (Fig. 3b) and 33 loci were associated in the recessive model (Fig. 3c; see Additional file 2: Table S3). The estimated heritability for TBRD was 0.42 ± 0.07. The top five loci for TBRD in both the additive and dominant models were shared (Fig. 2b) and contained eight positional candidate genes but these loci contained no TFBS (Table 1). The five most significant loci in the recessive model contained eight positional candidate genes and two TFBS. In all, 125 additional positional candidate genes were identified in the remaining 134 loci associated with TBRD from additive, dominant and recessive models.

After the GWAA were performed, significant loci associated with CCR1 and TBRD were compared to identify if any loci were shared. In total, 122 loci were shared across phenotypes and models (Fig. 2c). This included four of the top five loci shared in the additive, dominant, and recessive models for CCR1 and TBRD (Table 1).

Eighty-three loci associated with CCR1 contained TFBS for 51 transcription factors (see Additional file 2: Table S2). This included one of the most significant loci association with the recessive model (Table 1). Many of the TFBS were shared in the loci associated with CCR1. For example, the TFBS for NF-1/L was present at 15 loci, STAT4 was present at 7 loci, HNF-3β was present at 7 loci, and Pax-5, Nrf2:Mafk, JunD and c/EBPα were each present at 4 loci.

Twenty-eight loci associated with TBRD contained TFBS for 27 unique transcription factors (see Additional file 2: Table S4). None of the five most significant loci associated with the additive, dominant, or recessive models contained TFBS. However, four transcription factors (NF-1/L, Nrf2:MafK, C/EBPα, JunB) had binding sites identified at multiple loci (see Additional file 2: Table S4).

### Copy number variants (CNVs)

Of the 282 unique loci associated with CCR1, 55 (19.4%) contained SNPs located within one or more CNVs identified in cattle (see Additional file 2: Table S1). When the TBRD loci were compared with CNV boundaries, 30 loci contained SNPs that were located within one or more CNVs which represents 22.4% of all of the unique loci associated with TBRD (see Additional file 2: Table S3). When the 122 shared loci associated with CCR1 and TBRD were compared, 26 (21.3%) were within a CNV (see Additional file 2: Table S1 and Table S3).

### Correlations to Milk yield

No significant correlations were identified between genotypic and phenotypic correlations between 305MY and CCR1 or 305MY and TBRD (*P* > 0.05). The genetic correlation between CCR1 and 305MY was − 0.046 ± 0.14 and the phenotypic correlation was − 0.0024 ± 0.031. Similarly, the genetic correlation between TBRD and 305MY was 0.11 ± 0.17 and was 0.043 ± 0.032 for the phenotypic correlation. The investigation of the pleiotropic effects of CCR1 and TBRD with 305MY identified no significant correlations.

## Discussion

### Heritability estimates

The heritability estimates for CCR1 and TBRD were higher than previously reported for fertility traits [14–20]. One exception was a recently published paper using a similar experimental design in Holstein heifers investigating loci associated with heifer conception rate [21], which reported a heritability of 0.61. These high heritability estimates are likely due to the experimental design. Cows genotyped in this study were not randomly sampled from the normal range of a fertility distribution but were chosen from the extreme tails of the distribution, resulting in a sampling bias. Further sampling bias resulted from eliminating cows with confounding factors such as lameness, disease, dystocia and other health factors previously identified to reduce fertility. The selection of these cows from the tails of the phenotypic distribution was designed to enhance the study with cows that were more likely to conceive or fail to conceive based on an underlying genetic predisposition. In previous studies, particularly those based on national or international data with tens or hundreds of thousands of animals, heritability estimates are not based on sampling cattle from extreme tails of the phenotypic distribution and cattle that exhibited confounding factors such as lameness, disease and dystocia were included in the population from which heritability was estimated. Studies based on national evaluations would also likely contend with more environmental variability and variations in phenotypic measurements than the smaller number of dairies involved in this study. Directly comparing heritability estimates for fertility traits in studies with differences in ascertainment should be done with caution, as the phenotypes, AI sires, population structures, and the herd management practices of the different study populations can be quite diverse. Although the sampling biases within this study may have resulted in inflated heritability estimate, this disadvantage may have been overcome by its theoretical advantage in detecting loci associated with fertility in a GWAA with fewer samples.

### Loci associated with both phenotypes

The measurement of CCR1 and TBRD provides an overview of the complex processes involved in fertilization, placental development, implantation, maternal recognition of pregnancy and early embryonic development. It is expected that some but not all loci associated with HCR1 would be the same as those associated with TBRD because of the complexity of the reproductive processes in the first month of gestation. However, the overlap of associated loci between models and across phenotypes suggests a common genetic regulation of both fertility measurements and the potential to improve both CCR1 and TBRD simultaneously with genomic selection. The most significant loci associated with CCR1 and TBRD will, therefore, be discussed further as these loci offer an insight into both CCR1 and TBRD.

Eight of the top 15 loci (top 5 from each model) were shared across phenotypes, including 4 loci shared between the additive and dominant models and 4 loci shared between the recessive models (Table 1). The function of the 12 overlapping positional candidate genes were similar, and could be placed into three groups based on their functions related to cell adhesion, olfactory receptors, and steroid hormones.

The four positional candidate genes associated with cell adhesion (armadillo repeat gene deleted in velocardiofacial syndrome (*ARVCF*), gap junction protein beta 4 (*GJB4*), gap junction protein beta 5 (*GJB5*), and junction mediating and regulatory protein, p53 cofactor (*JMY*)) are of interest as the function of cell-cell junctions must change during early pregnancy establishment [22]. The *ARVCF* gene is a member of the catenin family with functions related to adherens junctions, which are cadherin-based adhesive structures that connect actin filaments between neighboring cells [23]. Adherens junctions are important regulators of uterine structure, and influence uterine receptivity to implantation in sheep [24]. The *JMY* gene produces proteins that are involved in the regulation of cadherins [25], which form adherens junctions, and are critical to pregnancy establishment [26]. Cadherins and adherens junctions have multiple functions during early pregnancy in sheep, as there is a decrease in adherens junctions between days 10–12 of gestation and then an increase in adherens junctions between days 14–16 [24]. The initial decrease in adherens junctions allows for an increased transudation that facilitates blastocyst elongation, while the increase in adherens junctions two days later facilitates implantation [24]. While *ARVCF* and *JMY* proteins have functions relating to adherens junctions, the gene products of *GJB4* and *GJB5* are important in gap junctions, which are intracellular ion channels that allow the passage and exchange of second messenger molecules and electrical impulses between the cytoplasm of two cells. The function of gap junctions as a means of communication between uterine stromal cells in early pregnancy is vital to uterine angiogenesis and embryo survival [27]. The *GJB4* and *GJB5* genes are differentially expressed depending on day of early pregnancy in placental trophoblast cells in mice [28] and in endometrial cells of pigs [29].

The second positional candidate gene group relates to olfactory receptors and contains three genes: olfactory receptor 8 U9-like (*LOC787620*), olfactory receptor-like protein OLF2 (*LOC787642*), and olfactory receptor-like protein OLF2 (*LOC787667*). Proteins encoded by these genes constitute olfactory receptors important in signaling pathways of the olfactory system [30, 31]. Olfactory receptors, are commonly expressed by sensory neurons contained in mammalian olfactory epithelium [32], and are involved in reproduction in many mammalian species [33–35] including cattle [36, 37]. However, several olfactory receptors are expressed in other tissues such as the uterus and the testis [38], although the functions of most olfactory receptors located outside of olfactory neurons remains unknown [39]. Olfactory proteins within the olfactory epithelium have roles in fertility because of the intimate relationship with the hypothalamic pituitary axis and the release of reproductive hormones such as gonadotropin-releasing hormone (GnRH), oxytocin and vasopressin [40, 41] which are important in preparation and maintenance of the uterus for pregnancy.

The final group of positional candidates contains zinc finger DHHC - type containing 21 (*ZDHHC21*) and catechol-O-methyltransferase (*COMT*) genes which have functions relating to estrogen and progesterone and/or their receptors which play an important role in preparing the uterus for and maintaining a pregnancy. This is the second study to find an association of *ZDHHC21* with fertility as *ZDHHC21* was also identified as associated with fertility in a study using a pathway analysis of genes in beef heifers subjected to serial embryo transfers [37]. The *ZDHHC21* gene produces a protein that functions as a palmitoyl - acyltransferase for estrogen receptor alpha, progesterone receptor, and the androgen receptor in mice [42]. The action of progesterone, facilitated by the progesterone receptor in the uterus, is critical for pregnancy success [43] as progesterone signaling modulates endometrial gene expression essential to embryonic development and pregnancy establishment [44, 45]. Estrogen and estrogen receptors are also important for a successful pregnancy as estrogen is essential for the preparation of the uterus for implantation. The positional candidate gene, *COMT,* is a critical component in estrogen metabolic pathways [46]. The activation of *COMT* during the estrous cycle and pregnancy has led researchers to speculate that *COMT* activity is sensitive to estrogen levels [47, 48]. In support of this, studies have linked mutations in *COMT* with an increased risk of endometrial cancer [49] and premature ovarian insufficiency [50] in humans. Mutations that alter the function of estrogen, progesterone, and their receptors have the potential to alter uterine receptivity and implantation [51, 52] leading to pregnancy loss prior to day 35 .

Transcription factor binding sites regulate gene expression and thus mutations at these sites may alter gene expression of positional candidate genes that they are near. When the TFBS were investigated for the loci associated with CCR1 and TBRD, C/EBPα, JunD, NF-1/L, and Nrf2:MafK were identified in 2 or more of the 26 shared loci. The sharing of TFBS at 26 loci suggests that there may be joint regulation of CCR1 and TBRD loci in fertility processes (see Additional file 2: Table S2 and Table S4). For example, C/EBPα has been linked to impaired fertility, likely through regulation of the lutenization and vascular cell development in C/EBPα/C/EBPβ knockout mice studies [53], and has been linked to placenta development in humans [54]. Similar to C/EBPα, the Nrf2:MafK heterodimer regulates placental development in rats [55]. For the developing embryo and placenta to continue, implantation must be successful. The process of implantation requires invasion of the endometrium and in a human study, JunD and other AP-1 family members were involved in trophoblast cell invasion of the endometrium during the implantation process [56]. Unlike the other transcription factors, NF-1/L is not highly characterized but is thought to function in a similar fashion to neurofibromin 1 (NF-1) which negatively regulates the RAS/MAPK signal transduction pathway which also includes the transcription factors C/EBPα, JunE, NF-1, and Nrf2:MAFK [57–62]. The RAS/MAPK signaling pathway is involved in cellular growth, division, and migration, tissue repair, and angiogenesis [63, 64]. All of these functions are critical for the early embryo to survive and develop during the first weeks of gestation. Proper placental angiogenesis is particularly important and highly regulated as it is crucial for embryo survival and pregnancy success [65]. Poor placental vasculature can inhibit the exchange of embryonic nutrients and waste leading to abnormal growth and/or development [65, 66]. Mutations that impact the function of these transcription factors have the potential to interrupt the normal RAS/MAPK signaling and the key functions necessary for placental and embryonic development.

### Loci within copy number variants

An unexpected feature of the loci associated with CCR1 and TBRD was the number of loci that identified within CNVs. Of the unique (unshared) loci associated with CCR1 and TBRD, 59 (19.9%) were located within CNVs (see Additional file 2: Table S1 and Table S3). These loci are characterized by a lack of supporting SNP “trees” in Fig. 1 and Fig. 3, which is not unexpected given that a significant portion of CNVs exhibit low LD with flanking markers (r^2^ < 0.8) [67–69]. Given that the estimated frequency of CNVs in cattle is 2 to 7% of the genome [70] this represents an over representation of the number of fertility loci that would be expected to be found in CNVs. Although others have identified CNVs associated with reduced reproductive performance in cattle, it has not been at this frequency. For example, Kadir et al. (2014) [71] identified a single CNV on BTA12 in Nordic Red cattle and McDaneld et al. (2014) [72] identified a single CNV on BTA5 in *Bos indicus* cattle that were associated with fertility. As this result was unexpected, the current study did not conduct an independent CNV analysis as the Illumina BovineHD BeadChip was not designed to have sufficient resolution to differentiate genotype intensity differences. Further studies are needed to determine the role of CNVs with cow fertility using genotyping methods that are specifically designed to detect CNVs.

### Correlation of loci associated with conception rate and Milk production

The selection for primarily milk production in dairy cattle in past decades has resulted in a decay of fertility until selection for fertility traits was included in multiple trait selection indexes in 2003 [12]. Whether this decay was due to a lack of selection for fertility traits or direct negative correlation with milk traits has been debated. To determine if the loci identified in this study were negatively correlated, the phenotypic and genetic correlations between 305MY, CCR1 and TBRD were investigated and found to be small and included zero within the bounds of their standard errors. This indicated that selection for fertility in this population would be unlikely to negatively impact milk production. These results differ from some previous studies where genetic correlations between fertility traits and milk production were unfavorable [73–76]. Differing management practices may have contributed to the alternate conclusions between studies [4]. For example, LeBlanc (2004) [77] reported that herds with milk production > 10,000 kg per lactation achieved higher fertility than lower producing herds, and the effect was largely due to superior reproductive and nutritional management practices. The LeBlanc [77] study stressed the importance of managing the nutritional requirements for high producing cows to meet the energy demands required for high fertility. Differences in nutritional and reproductive practices between the current and previous studies might have contributed to the correlation differences between the traits.

### Comparison of loci and positional candidate genes identified in previous studies as associated with fertility

Identifying loci that were associated with CCR1 and TBRD with other cattle fertility studies provides support for further investigation of these loci for genomic selection, to identify causal mutations, and to further understand their role in the complex processes that occur in the first month of gestation. The loci associated with CCR1 and TBRD were compared to 22 previous fertility studies in dairy and beef cattle (Table 2). Thirty-six loci associated with CCR1 and/or TBRD were identified in other studies including seven that have been identified in two or more studies (Table 2). These loci validated in multiple breeds (Holstein [21, 78–81], Jersey [80] and Angus [37]) and across life stages (Table 2). Additionally, loci from this study were compared to previously identified fertility haplotypes in Holsteins [82]. One QTL (*rs134964346*) identified in the current study, fell within Holstein haplotype HH5, which is located on BTA9 between 93,232,651 and 93,370,998 bp. This haplotype is associated with premature pregnancy termination prior to day 60 of gestation and has a carrier frequency of approximately 5% in North American Holsteins [83]. The identification of loci across independent populations, within and across breeds, suggests that the associated SNPs are located in close proximity to the causal variant and have large common effects on fertility. Identifying causal variants associated with fertility traits would allow the cattle industry to make significant genetic improvement without the need to continuously assess the usefulness of markers in LD with the causal variant.

**Table 2:** Loci associated with cattle fertility across studies

BTA^1^	Region (Mb)^2^	Previous Study(s)^3^	Previous Phenotype(s)^4^	Previous Breeds^5^	Current Study Phenotype(s)^6^
1	16–17	78	AISC	Holstein^N^	CCR1
1	25–26	81	DPR	Holstein^U^	CCR1
1	62–30	78	AISC	Holstein^N^	CCR1 & TBRD
1	83–84	78	AISH	Holstein^N^	CCR1 & TBRD
1	86–87	78	AISC	Holstein^N^	CCR1
2	53–54	78	AISC	Holstein^N^	CCR1
2	123–124	21,78	HCR1 & TBRD[21]; AISH[78]	Holstein^N,U^	CCR1
3	72–73	78	AISC	Holstein^N^	CCR1 & TBRD
3	81–82	78	AISC	Holstein^N^	TBRD
3	98–99	21	HCR1 & TBRD	Holstein^U^	CCR1 & TBRD
4	37–38	21	HCR1 & TBRD	Holstein^U^	CCR1 & TBRD
5	21–22	21	TBRD	Holstein^U^	CCR1
5	36–37	21	HCR1	Holstein^U^	CCR1 & TBRD
6	38–39	37	P28	Angus crosses^U^	CCR1 & TBRD
6	93–94	21,78	HCR1 & TBRD[21]; AISC[78]	Holstein^N,U^	CCR1
8	20–21	78	AISC	Holstein^N^	CCR1 & TBRD
8	24–25	78	AISC & AISH	Holstein^N^	CCR1
9	87–88	78	AISC	Holstein^N^	CCR1
11	16–17	21	HCR1 & TBRD	Holstein^U^	CCR1 & TBRD
11	77–78	78	AISH	Holstein^N^	CCR1 & TBRD
11	86–87	21	HCR1 & TBRD	Holstein^U^	CCR1
13	29–30	78,79	AISH[78]; ICF[79]	Holstein^C,N^	CCR1
15	26–27	21	HCR1 & TBRD	Holstein^U^	CCR1 & TBRD
17	3–4	78, 80	FTI, IFLC[78]; CI[80]	Holstein^A,I,N^, Jersey^A^	CCR1
17	74–75	21	HCR1 & TBRD	Holstein^U^	CCR1
17	75–76	21	HCR1 & TBRD	Holstein^U^	CCR1 & TBRD
18	8–9	78	AISC	Holstein^N^	TBRD
18	21–22	21	HCR1 & TBRD	Holstein^U^	CCR1
19	7–8	21	HCR1 & TBRD	Holstein^U^	CCR1
20	27–28	21,78	TBRD[21]; AISC[78]	Holstein^N,U^	CCR1 & TBRD
24	25–76	78	AISC	Holstein^N^	CCR1
26	19–20	78	AISC	Holstein^N^	CCR1
26	28–29	21,78	HCR1 & TBRD[21]; AISC[78]	Holstein^N,U^	CCR1
26	40–41	81	DPR	Holstein^U^	CCR1
27	21–22	21	HCR1 & TBRD	Holstein^U^	CCR1 & TBRD
28	29–30	78, 80	NRRH[78]; CI[80]	Holstein^A,I,N^, Jersey^A^	CCR1

^1^Bos taurus chromosome (BTA) location of the locus

^2^Region associated locus is located in (in Mb) as measured by numbered nucleotides in reference to the UMD 3.1 genome assembly (http://bovinegenome.org/?q=node/61; accessed 15 September 2016)

^3^The citation number for each study a locus was previously associed in is listed

^4^Traits previously associated with loci abbreviated as follows: AISC - number of inseminations to conception in cows; AISC - number of inseminations to conception in heifers; CI - calving interval; HCR1 - conception rate to first insemination in heifers; DFS - days to first service; DPR - daughter pregnancy rate; FTI - fertility index; HCR - heifer conception rate; ICF - interval (in days) from calving to first insemination; IFLC - days from first to last insemination in cows; NRRC - 56 day non return rate in cows; NRRH - 56 day non return rate in heifers; P28- pregnancy success at day 28 post embryo transfer; P42 - pregnancy success within first 42 days of mating; TBRD - number of times bred to conception. If multiple traits are listed from different studies the citation number for study is listed in superscript brackets

^5^Cattle breeds loci were previously identified are listed with the country or region the population was from indicated in superscript as follows: Australia - A; Canada - C; Ireland - I; Nordic - N; United States - U

^6^Phenotype of the current study the loci was associated with: conception rate at first AI service - CCR1 and TBRD

In addition to comparing the loci identified in this study with previous studies, positional candidate genes were investigated to identify if they have demonstrated to be differentially expressed during pregnancy or in the uterus of fertility classified cattle in previous studies. Positional candidate genes identified in this study were compared to two previous studies that investigated differential expression of genes in fertility classified beef heifers [84, 85]. There was no concordance in the positional candidate genes in the current study and the genes differentially expressed in the Geary et al. (2016) study [84]. However, 53 (of 291) positional candidates identified in this study were identified as differentially expressed in a study by Moraes and colleagues (2018) [85]. Fifteen of the positional candidate genes associated with CCR1 or TBRD were differentially expressed in multiple fertility comparisons (see Additional file 2: Table S5). Many of these genes have been linked to fertility through their roles in trophoblasts (*GJB5* [86], *NOD1* [87], *ROBO1* [88]), decidulization (*NDRG3* [89], and *NOTCH2* [90]), hormone regulation (*PTGFRN* [91]) and uterine pH (*CA12* [92]). Mutations that alter the functions of these genes have the potential to impair cellular communication, implantation, and create an unfavorable uterine environment which could contribute to early pregnancy loss.

## Conclusion

The loci and positional candidate genes associated with CCR1 and TBRD identified in this study provide further data for use in genomic selection of dairy cattle. Additionally, the loci associated with favorable CCR1 and TBRD were not found to be negatively correlated with 305MY, indicating that selection using these loci would not impair milk production which is of particular importance in the dairy industry.

Multiple loci identified in this study have positional candidate genes with functional relevance to CCR and have been previously tied to fertility in dairy and beef cattle. The validation of the fertility loci in multiple breeds indicates that these loci have large effects on fertility and may be used to enhance fertility across breeds. Further characterizations of regions associated with fertility across populations is needed to identify the causal mutations that are associated with fertility. The identification of causal mutations will enhance the accuracy of genomic selection for CCR1 and TBRD and will aid in the understanding of the mechanisms responsible for successful pregnancy in contrast to early embryonic loss.

## Methods

### Study population and phenotypes

This study was conducted with the approval of the Institutional Animal Care and Use Committee at Washington State University (4295). Holstein cows (*n* = 2015) from six dairy operations (Cow Palace, DeRuyter Brothers Dairy, Five D Dairy, George DeRuyter Dairy, J&K Dairy, and Sunnyside Dairy) located in central Washington were followed to determine CCR. Only primiparous cows were evaluated and enrolled in this study. Cows received artificial insemination (AI) upon observed estrus or at timed AI. Pregnancy status was determined 35 days after AI by rectal palpation of the uterus. DairyComp 305 (Valley Agricultural Software, Tulare, CA) records were used to remove cows from the study that suffered from any ailment that might have an effect on fertility. These ailments included: abortions, dystocia, uterine diseases, fever, foot disease, mastitis, metabolic issues, pink eye, and respiratory disease. After censoring cows with health issues, 1064 cows were selected for genotyping. The cows that were genotyped included approximately equal proportions of the tails of the phenotypic distribution represented by highly fertile (*n* = 498) and subfertile or infertile cows (*n* = 566). Highly fertile cows conceived at the first AI, whereas subfertile cows conceived on or after the fourth AI service and infertile cows were those that failed to conceive after six or more AI attempts. Subfertile and infertile cows were inseminated 4 to 20 times (see Additional file 1: Figure S1). As infertile cows did not become pregnant, they were excluded from the TBRD analysis. The fertility phenotypes CCR1 and TBRD were based on successful maintenance of a pregnancy to day 35 post-AI.

Artificial insemination was performed (depending on individual dairy practices) by one of 34 technicians to one or more sires. Conception rate of cows did not differ between AI technicians (*P* > 0.05). Frozen-thawed semen from 433 Holstein and 2 Angus sires was used for AI, with a mean conception rate (CR) for all sires of 26.8%. No sexed semen, which could have an impact on conception rate, was utilized in the current study. The CR between sires was not different (*P* = 0.99) within or between breeds, therefore AI sire was not included as a covariate in the model. Cows that did not conceive to the first AI service were usually rebred to different AI sires at each additional service, although this was dependent on individual dairy practices. Breeding cows to different AI sires at each service reduced the possibility that a cow’s failure to conceive was due to her being bred to a subfertile or infertile bull. Health and milk production data were collected through DairyComp 305 (Valley Ag Software, Tulare, CA) to determine if CCR1 and TBRD were correlated with milk production.

### DNA extraction and genotyping

Whole blood (~ 16 ml) was collected into EDTA tubes from cows via venipuncture of the tail vein. The DNA was extracted from white blood cell pellets using the Puregene DNA extraction protocol as per manufacturer’s instructions (Gentra, Minneaplois, MN). After extraction, DNA was quantified with a NanoDrop 1000 spectrophotometer (ThermoFisher Scientific, Wilmington, DE) and genotyped at Neogen Laboratories (Lincoln, NE) using the Illumina (San Diego, CA) BovineHD BeadChip. The BovineHD BeadChip contains 778,962 SNPs with an average distance between SNPs of 3.43 kb [93].

### Quality control

Prior to the GWAA, 11 cows were removed for quality control due to a low genotyping call rate (< 0.90), and 21 cows were removed for being turned out with a bull to receive a natural service rather than AI. SNPs underwent quality control for a low genotyping call rate (< 0.90; 10,421 SNPs removed), a low minor allele frequency (< 0.01; 142,539 SNPs removed), and a failure of SNPs to be in Hardy-Weinberg equilibrium (*P* < 10^− 100^; 109 SNPs removed). After quality control, 625,093 SNPs and 1032 cows (494 highly fertile and 538 subfertile or infertile) remained for the CCR1 analysis. For the TBRD analysis, 966 cows (494 highly fertile and 472 subfertile) remained after quality control (see Additional file 1: Figure S1).

### Genome-wide association analysis

The GWAA were performed for CCR1 and TBRD using an efficient mixed-model association eXpeditied (EMMAX) model [94] in the SNP and Variation Suite (SVS) software (version 9.1) (Golden Helix, Bozeman, MT; http://goldenhelix.com/products/SNP_Variation/index.html) [95]. The general mixed model is described as ***y*** = **Xβ** ***+ Z****u* + ϵ, where **y** explains the *n* × 1 vector of observed phenotypes, **X** is an *n* × *f* matrix of fixed effects (*f*), **β** is an *f* × 1 vector containing the fixed effect coefficients, and **Z** is an *n* × *t* matrix relating the random effects (*t*) to the phenotype, and *u* is the random effect of the mixed model [96]. The model assumes residuals to be independent with an identical distribution such that *Var*(*u*) = *σ*_*g*_^2^***K*** and (*ϵ*) = *σ*_*e*_^2^***I***, and *Var*(*y*) = *σ*_*g*_^2^***ZKZ***^′^ + *σ*_*e*_^2^***I*.** For this study **K** is a matrix of pairwise genomic relationships and **Z** is the identity matrix, **I** [96].

Since the exact mode of inheritance for CCR1 and TBRD is unknown and may not be strictly additive, three genotypic models (additive, dominant, and recessive) were analyzed for each phenotype. In the additive model associations with fertility assumes two minor alleles (aa) resulted in twice the effect on fertility as a single minor allele (Aa). Association with fertility in the dominant model is determined by comparing the presence of at least one minor allele (Aa or aa) to no minor alleles (AA), whereas the recessive model compared the presence of two minor alleles (aa) with at least one major allele (AA or Aa) as previously described http://doc.goldenhelix.com/SVS/latest/svsmanual/genotype_association_tests.html.

EMMAX estimated pseudo-heritability using the equation ${h}^2=\frac{\upsigma_g^2}{\upsigma_g^2+{\upsigma}_e^2}$ in SVS, were ${\upsigma}_g^2$ is the additive genetic variance and ${\upsigma}_e^2$ is the environmental variance [96]. However, pseudo- heritability can be over-inflated when estimated with EMMAX in SVS with small sample sizes. Given this, the heritability estimates for CCR1 and TBRD for this study were instead calculated in SVS with a genomic best linear unbiased predictor (GBLUP) analysis [97] using the average information algorithm (AI-REML), which is a bivariate restricted maximum likelihood analysis [98, 99]. The AI-REML GBLUP method is commonly used for calculating heritability, although it is done at the expense of increased computational time. Further documentation of SVS methods for EMMAX, pseudo-heritability and GBLUP with AI-REML are available (http://doc.goldenhelix.com/SVS/latest/svsmanual/mixedModelMethods/overview.html).

To determine if loci were associated with CCR1 or TBRD, a genome-wide significance threshold for unadjusted *P*-values of *P* < 5.0 × 10^− 08^ was used based on recommendations by the International HapMap Consortium [100, 101]. To identify boundaries of a locus, any SNP in linkage disequilibrium (LD; D’ > 0.7) with a SNP associated with fertility was considered to comprise the same locus. The D’ threshold falls within previous thresholds reported to characterize SNPs within a locus [102–105]. Positional candidate genes were identified within a 34 kb region surrounding significant SNPs (17 kb 5′ and 3′ of associated SNPs) based on the average haplotype block size in Holstein cattle estimated using the method previously described by Gabriel et al. (2012) in SVS [106]. Additionally, SNPs were investigated in the Ensembl database [107] to determine if they were located within the defined boundaries of copy number variants (CNV).

### Transcription factor binding sites

Putative TFBS influenced by the allele present at loci associated with CCR1 or TBRD were identified using PROMO, a virtual laboratory used to query putative TFBS [108, 109]. PROMO utilizes the TRANSFAC transcription factor database [110] to identify TFBS and calculate the probability of a TFBS within a specific DNA sequence by generating a test statistic called a random expectation (RE) query [108, 109]. The TRANSFAC databased was searched for 31 bp sequence (15 bp before and after the associated SNP) that included both SNP alleles to identify TFBS. Significant TFBS were required to have a RE query value < 0.05, span the SNP of interest, and only be present with one of the two alleles of the associated SNP.

### Genetic and phenotypic correlations to Milk yield

To understand the potential impact of genomic selection for fertility traits on milk production, genetic and phenotypic correlations between CCR1, TBRD, and 305-day milk yield (305MY) (kg) were computed. Records of 305MY were obtained for each cow’s first lactation using DairyComp 305. Genetic correlations were computed in SVS using a GBLUP analysis [95] with the AI-REML algorithm [98, 99] and a genomic relationship matrix to find the additive genetic variance for each trait and the additive genetic covariance between either CCR1 and 305MY or TBRD and 305MY. The resulting variances and covariance were used to calculate a Pearson’s correlation coefficient and standard error between the fertility trait and 305MY. For these analyses, dairy was a covariate. Phenotypic correlations were computed in R Studio 1.0.153 [111] using R version 3.0.2 [112] as partial correlations to control for the effect of dairy on each trait.

In addition to calculating the genotypic and phenotypic correlations between fertility and milk production, each SNP associated with CCR1 or TBRD was investigated for pleiotropic effects on milk production using a one-way analysis of variance for milk production (305MY) between genotypes. A Bonferroni multiple testing correction threshold was used to identify pleiotropic effects of CCR1 (*P* < 0.0001) and TBRD (*P* < 0.0004) with 305MY.

## Supplementary information

**Additional file 1: Figsure S1.** Breakdown of number of times bred for subfertile/infertile cows pre- and post-quality control (QC).

**Additional file 2: Table S1.** Loci associated with cow conception rate at the first breeding. **Table S2.** Loci associated with conception rate at first breeding containing transcription factor binding sites. **Table S3.** Loci associated with number of times bred to conception. **Table S4.** Loci associated with number of times bred to conception containing transcription factor binding sites. **Table S5.** List of positional candidate genes found to be differentially expressed by Moraes et al., (2018).

## Data Availability

The data used and analyzed in the current study are available from the corresponding author on reasonable request.

## Abbreviations

305MY: 305-day milk yield
AI: Artificial insemination
AI-REML: Average information algorithm restricted maximum likelihood
CCR: Cow conception rate
CCR1: Cow conception rate at first service
CNV: Copy number variation
EMMAX: Efficient mixed model expedited
GBLUP: Genomic best linear unbiased predictor
GWAA: Genome-wide association study
LD: Linkage disequilibrium
SVS: SNP and variation suite
TBRD: Number of times bred to conception
TFBS: Transcription factor binding sites
