# Supplementary figures and images for: Identification of loci associated with conception rate in primiparous Holstein cows
Source: BMC Genomics. 2019 Nov 12;20:840. doi: 10.1186/s12864-019-6203-2 (PMC6852976; doi:10.1186/s12864-019-6203-2)

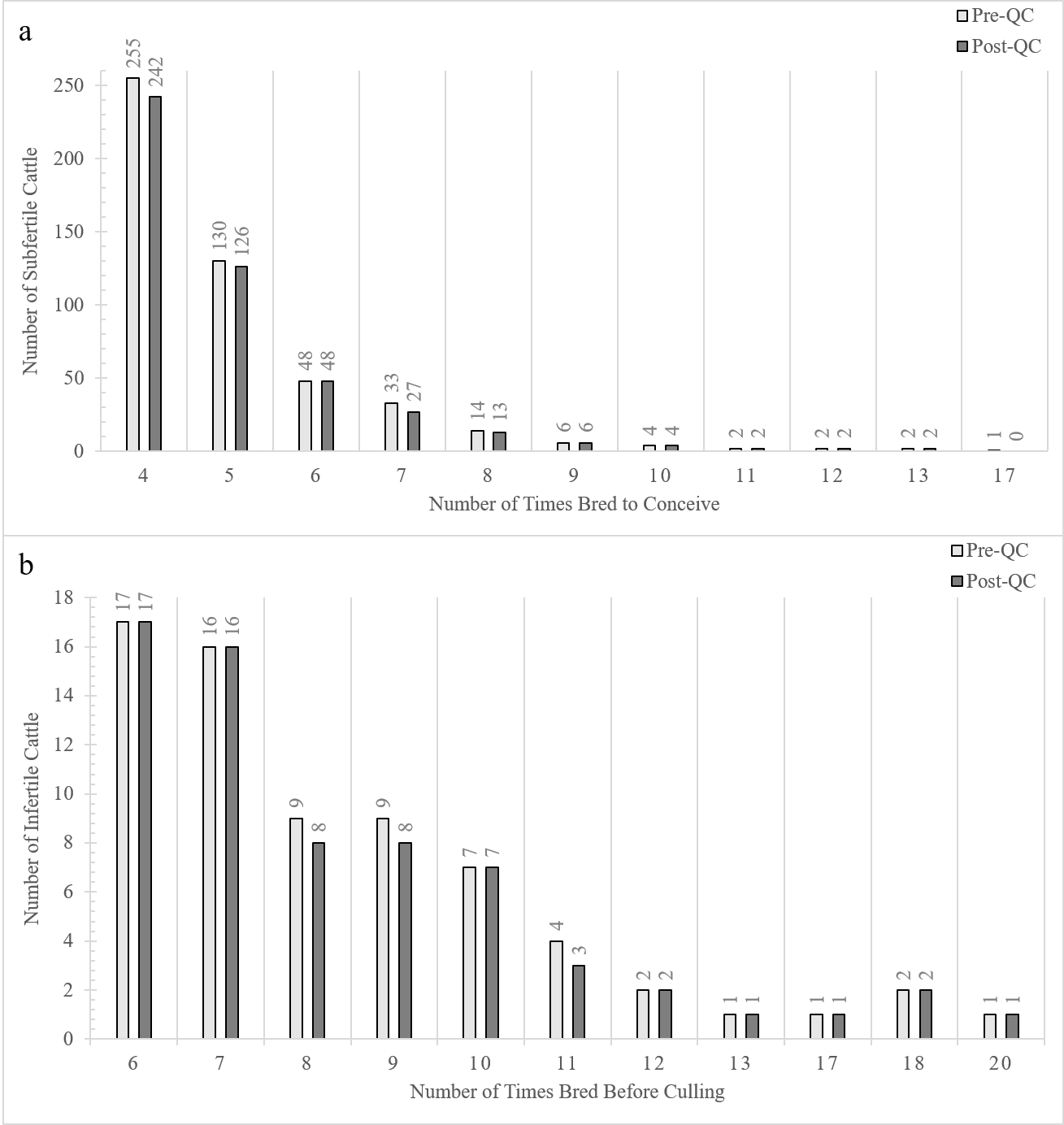

Supplement: Supplementary file 1 — Additional file 1: Figsure S1. Breakdown of number of times bred for subfertile/infertile cows pre- and post-quality control (QC). [file 12864_2019_6203_MOESM1_ESM.tif]
