# Supplementary material for: Identification of loci associated with conception rate in primiparous Holstein cows
Source: BMC Genomics. 2019 Nov 12;20:840. doi: 10.1186/s12864-019-6203-2 (PMC6852976; doi:10.1186/s12864-019-6203-2)
Supplement: Supplementary file 2 — Additional file 2: Table S1. Loci associated with cow conception rate at the first breeding. Table S2. Loci associated with conception rate at first breeding containing transcription factor binding sites. Table S3. Loci associated with number of times bred to conception. Table S4. Loci associated with number of times bred to conception containing transcription factor binding sites. Table S5. List of positional candidate genes found to be differentially expressed by Moraes et al., (2018). [file 12864_2019_6203_MOESM2_ESM.pdf]

Table S1. Loci associated with cow conception rate at the first breeding.

BTA[1]	ARS 1.2 BP Position[2]	UMD 3.1 Position[3]	SNP ID[4,5]	Model[6]	P-Value[7]
UK	UK	BTAX 123,316,017	*rs134099044*	Additive<br>Dominant	2.75E-09<br>2.75E-09
1	716,721	36,337	*rs132895573**	Recessive	2.16E-09
1	17,190,292	16,670,513	*rs136034629**	Recessive	1.52E-08
1	26,123,942	25,626,521	*rs43222718*	Dominant	3.43E-09
1	33,136,647	32,700,496	*rs43226666**	Additive<br>Dominant	4.55E-10<br>2.84E-13
1	33,522,801	33,086,024	*rs43226580**	Dominant	1.71E-12
1	36,101,420	35,698,088	*rs43226992*	Additive	3.96E-09
1	61,470,011	62,056,267	*rs42760220*	Additive<br>Dominant	1.47E-09<br>2.13E-10
1	72,665,912	73,273,238	*rs137394358**	Dominant	2.07E-08
1	82,457,094	83,047,838	*rs109289667*	Recessive	1.10E-09
1	84,573,157	85,187,247	*rs43247724*	Additive<br>Dominant	2.45E-08<br>2.45E-08
1	85,375,697	86,006,808	*rs29025180*	Dominant	2.63E-08
1	98,891,232	99,689,088	*rs110245908*	Dominant	4.09E-09
1	99,081,740	99,879,710	*rs137156811*	Additive<br>Dominant	3.63E-21<br>3.63E-21
1	113,023,021	113,931,474	*rs42215584*	Additive<br>Dominant	3.97E-19<br>7.54E-21
1	123,269,176	124,246,283	*rs41575917*	Recessive	1.66E-09
1	127,938,641	128,938,068	*rs43265135**	Additive<br>Dominant	1.05E-10<br>2.62E-13
1	138,454,971	139,950,787	*rs43278206*	Dominant	5.54E-10
1	141,690,171	143,188,538	*rs134923101*	Recessive	1.92E-10
1	155,709,024	157,448,783	*rs43285260*	Recessive	1.51E-09
2	4,950,986	4,883,261	*rs108949806*	Dominant	1.79E-10
2	13,489,742	13,518,945	*rs137280033*	Additive<br>Dominant	1.65E-09<br>1.65E-09
2	33,584,278	33,688,017	*rs110717447*	Recessive	2.93E-09
2	53,451,414	53,552,010	*rs42534341*	Additive	1.26E-08
2	76,334,435	76,755,188	*rs134705498*	Additive<br>Dominant	9.19E-10<br>1.08E-09
2	86,075,750	86,479,252	*rs133738177*	Additive<br>Dominant	2.71E-23<br>2.71E-23
2	98,041,003	98,468,702	*rs136360354*	Dominant	1.49E-08
2	123,224,834	123,768,735	*rs41636509**	Additive<br>Recessive	4.16E-08<br>9.47E-12
2	123,403,424	123,947,527	*rs110886070**	Dominant	1.11E-08
2	129,277,519	129,876,162	*rs136809757**	Additive<br>Dominant	1.28E-11<br>1.28E-11

3	11,494,736	11,612,564	*rs110900677*	Additive	1.63E-08
3	16,594,030	16,653,795	*rs133063046*	Dominant	6.79E-10
3	23,385,126	23,494,800	*rs134056476*	Additive<br>Dominant	6.56E-18<br>1.78E-18
3	26,320,263	26,436,103	*rs109659431*	Additive<br>Recessive	8.85E-10<br>4.61E-10
3	31,645,740	31,800,315	*rs43746101*	Additive<br>Recessive	7.84E-09<br>8.27E-12
3	54,398,276	54,575,757	*rs110480314**	Dominant	2.03E-11
3	72,002,117	72,296,340	*rs29013285*	Dominant	4.54E-10
3	76,838,701	77,163,315	*rs136533238*	Dominant	1.80E-10
3	97,093,984	97,680,312	*rs41585055*	Additive<br>Dominant	1.55E-11<br>2.93E-16
3	97,504,864	98,096,750	*rs29024665*	Dominant	6.41E-12
3	97,690,487	98,284,456	*rs43360769*	Recessive	5.45E-11
3	110,948,044	111,540,905	*rs133945887*	Recessive	1.33E-14
3	116,861,907	117,489,001	*rs43371109*	Additive<br>Dominant	3.17E-09<br>9.20E-13
4	4,788,623	4,700,972	*rs136994488*	Recessive	2.27E-08
4	9,919,989	9,772,807	*rs137111726*	Dominant	5.89E-09
4	37,056,340	37,235,676	*rs133417267*	Additive<br>Dominant	2.93E-13<br>2.93E-13
4	43,184,336	43,419,900	*rs109009636*	Recessive	2.25E-10
4	44,864,073	45,128,631	*rs13665021*	Dominant	1.81E-08
4	48,440,026	48,718,180	*rs136800119**	Additive<br>Dominant	1.43E-08<br>1.43E-08
4	55,979,237	55,988,253	*rs132801901*	Dominant	2.47E-11
4	60,044,804	60,433,843	*rs133973179*	Additive	7.97E-09
4	65,833,048	66,232,207	*rs108957740*	Dominant	6.22E-10
4	84,353,274	85,102,727	*rs135837483**	Additive	1.68E-08
4	92,464,322	93,277,289	*rs109721084*	Additive<br>Dominant	4.18E-11<br>3.75E-13
5	4,361,199	4,346,709	*rs110152722**	Additive<br>Dominant	1.43E-11<br>9.65E-12
5	12,616,464	12,688,864	*rs136793667*	Additive<br>Dominant	4.04E-12<br>1.28E-08
5	17,857,463	17,922,992	*rs132637832*	Additive<br>Dominant	2.23E-12<br>2.23E-12
5	21,290,477	21,357,397	*rs137251811*	Dominant	7.64E-10
5	28,061,170	28,218,085	*rs135989081*	Dominant	1.31E-10
5	28,774,907	28,939,485	*rs134199916**	Additive<br>Dominant	2.94E-12<br>2.94E-12
5	30,438,787	30,608,814	*rs133335343**	Additive<br>Dominant	1.60E-12<br>1.60E-12
5	36,663,436	36,860,611	*rs136252597*	Additive	1.01E-13

				Dominant	1.01E-13
5	40,344,343	40,576,651	*rs43434026*	Recessive	1.30E-08
5	45,981,588	46,183,975	*rs136476902*	Dominant	4.50E-08
5	58,573,797	58,906,988	*rs137278465**	Recessive	4.96E-08
5	59,146,023	59,503,184	*rs133515825**	Dominant	6.64E-09
5	59,553,709	59,905,836	*rs137261569**	Additive<br>Dominant	1.82E-25<br>1.82E-25
5	102,233,026	102,693,737	*rs109344731**	Dominant	7.77E-14
5	110,537,227	111,051,266	*rs136545177**	Recessive	9.29E-11
5	119,424,101	121,044,113	*rs133051543**	Recessive	3.20E-11
6	7,448,715	8,628,487	*rs136663821*	Dominant	1.24E-10
6	10,139,671	11,301,952	*rs29019244*	Dominant	9.31E-09
6	10,734,724	11,898,659	*rs137187491*	Additive<br>Dominant	4.73E-10<br>4.73E-10
6	17,693,932	18,946,287	*rs43456078*	Recessive	3.08E-09
6	30,369,047	31,792,754	*rs135006577*	Additive<br>Dominant	3.83E-24<br>3.83E-24
6	37,013,919	38,447,022	*rs109381958**	Additive<br>Dominant	4.31E-30<br>4.31E-30
6	45,218,126	46,763,699	*rs137502911*	Recessive	1.43E-09
6	53,086,414	54,689,211	*rs42762416*	Additive<br>Dominant	6.94E-15<br>6.94E-15
6	73,255,115	74,910,779	*rs110648584*	Additive<br>Dominant	3.35E-09<br>1.79E-10
6	79,147,024	80,854,287	*rs109934822**	Additive<br>Recessive	1.72E-09<br>6.06E-12
6	84,673,257	86,399,380	*rs132748387*	Additive<br>Dominant	8.86E-09<br>8.86E-09
6	91,512,574	93,263,835	*rs43476570*	Dominant	3.51E-08
6	91,908,296	93,660,111	*rs132854961*	Recessive	1.56E-14
6	98,510,667	100,293,854	*rs109245852*	Recessive	1.28E-10
6	98,949,730	100,733,028	*rs43488004*	Additive<br>Dominant	5.57E-17<br>3.38E-21
6	100,626,371	102,407,680	*rs134284115*	Recessive	6.91E-10
7	1,149,677	1,053,454	*rs133295796*	Additive<br>Dominant	8.66E-25<br>8.66E-25
7	7,183,086	8,207,142	*rs134787463**	Dominant	2.54E-09
7	7,199,859	8,223,914	*rs133396251**	Additive<br>Dominant	4.97E-11<br>9.22E-13
7	36,068,330	37,422,936	*rs132666128*	Recessive	3.10E-09
7	43,772,010	45,391,177	*rs110014023**	Recessive	4.65E-10
7	78,348,022	80,634,324	*rs132636869*	Dominant	1.76E-11
7	79,449,880	81,737,860	*rs133675138*	Additive	1.62E-08
7	96,416,881	98,828,347	*rs137216406*	Dominant	3.80E-10
7	97,540,689	99,952,417	*rs110327822*	Additive	7.30E-09

				Dominant	9.48E-14
8	20,675,446	20,685,523	*rs109551298*	Recessive	1.16E-10
8	20,721,025	20,731,035	*rs43539285*	Additive<br>Dominant	3.16E-13<br>6.84E-13
8	24,555,128	24,520,365	*rs134954592*	Dominant	1.14E-08
8	29,565,411	29,615,889	*rs134405734*	Additive<br>Dominant	1.42E-36<br>1.42E-36
8	47,118,716	47,416,086	*rs133499631*	Additive<br>Dominant	5.03E-09<br>5.03E-09
8	49,974,619	50,283,891	*rs109254920*	Dominant	1.45E-08
8	50,255,926	50,571,006	*rs134144724*	Additive<br>Dominant	9.92E-10<br>9.92E-10
8	58,921,513	59,331,066	*rs136685338*	Additive<br>Dominant	7.61E-10<br>1.77E-10
8	65,837,664	66,316,372	*rs133697170*	Additive<br>Dominant	1.51E-19<br>1.51E-19
8	76,695,824	78,049,107	*rs41859655*	Dominant	6.31E-11
8	77,981,766	79,362,912	*rs134979587*	Additive<br>Dominant	1.68E-14<br>1.68E-14
8	92,765,998	94,405,438	*rs43569214*	Additive	1.64E-08
8	101,333,339	103,040,511	*rs110198832*	Recessive	2.48E-08
8	102,293,921	103,998,687	*rs42359237*	Dominant	1.12E-08
8	111,550,769	UK	*rs134584163*	Additive<br>Dominant	2.93E-19<br>2.93E-19
9	6,538,240	6,661,699	*rs110968046**	Recessive	3.30E-09
9	43,839,708	44,351,018	*rs110310103*	Additive<br>Dominant	3.26E-10<br>4.54E-12
9	64,893,414	65,812,782	*rs110885320*	Recessive	1.83E-08
9	74,687,743	75,695,000	*rs134083077*	Additive<br>Dominant	1.36E-17<br>1.36E-17
9	83,719,602	84,824,332	*rs109177941*	Dominant	2.66E-09
9	86,166,629	87,314,566	*rs133550131*	Additive<br>Dominant	2.71E-08<br>4.81E-09
9	86,827,054	87,975,311	*rs134079040**	Additive<br>Dominant	1.95E-11<br>1.95E-11
9	91,931,462	93,306,447	*rs134964346*	Dominant	3.64E-08
9	97,468,572	98,925,099	*rs135920187*	Recessive	4.20E-08
10	34,950,052	35,050,522	*rs110393438*	Dominant	7.06E-09
10	43,101,892	43,145,489	*rs109533748*	Additive<br>Dominant	6.04E-11<br>6.04E-11
10	46,666,450	46,723,072	*rs133481026*	Additive<br>Dominant	4.99E-09<br>4.99E-09
10	62,992,388	63,217,046	*rs133388647*	Recessive	1.47E-12
10	67,250,158	67,481,157	*rs29026382*	Recessive	4.10E-13
10	76,396,374	76,708,701	*rs132793686*	Dominant	2.05E-09

10	80,522,688	80,873,088	*rs110456535*	Dominant	2.02E-11
10	87,401,016	88,409,131	*rs43026102*	Recessive	1.49E-08
10	91,225,759	92,349,953	*rs134880886*	Additive<br>Dominant	4.04E-09<br>2.30E-11
11	2,150,214	2,115,725	*rs110303601*	Dominant	1.51E-09
11	13,613,210	13,625,673	*rs43661407*	Recessive	3.39E-12
11	13,798,421	13,809,204	*rs110462022*	Dominant	8.46E-09
11	16,856,558	16,878,438	*rs109265815*	Dominant	1.48E-08
11	22,761,817	22,794,712	*rs109465638*	Recessive	2.50E-08
11	27,428,692	27,263,096	*rs137239134*	Recessive	4.28E-09
11	44,861,152	44,723,154	*rs136865423**	Recessive	6.99E-09
11	48,485,035	48,355,243	*rs134197588*	Recessive	6.84E-09
11	52,376,018	52,289,973	*rs136569977*	Dominant	4.30E-11
11	65,376,428	65,350,346	*rs110844122*	Dominant	1.01E-08
11	72,458,410	72,447,958	*rs134695420*	Additive<br>Dominant	3.93E-11<br>3.93E-11
11	77,409,930	77,475,988	*rs109117330*	Recessive	7.43E-10
11	81,737,489	81,885,400	*rs133374668*	Dominant	2.00E-09
11	86,434,490	86,400,864	*rs110541854*	Dominant	1.65E-08
11	92,127,718	92,107,430	*rs110293128**	Additive<br>Dominant	2.51E-08<br>2.89E-09
11	106,236,174	105,879,823	*rs136006377*	Additive<br>Dominant	2.48E-08<br>2.48E-08
12	3,950,729	3,902,971	*rs42273278*	Additive<br>Dominant	1.31E-10<br>1.31E-10
12	11,232,062	11,261,415	*rs134036785*	Additive<br>Recessive	1.42E-09<br>1.85E-09
12	13,263,086	13,305,470	*rs134850048*	Dominant	1.26E-08
12	14,179,880	14,220,951	*rs42421894*	Dominant	7.32E-09
12	22,847,412	22,868,176	*rs134779941*	Dominant	5.35E-09
12	55,301,894	55,644,058	*rs133586046*	Recessive	4.50E-09
12	62,999,694	63,506,888	*rs42401835**	Additive<br>Dominant	8.64E-14<br>8.64E-14
12	79,801,920	83,789,534	*rs133844853*	Dominant	7.16E-09
12	82,937,444	86,947,480	*rs136060818*	Dominant	1.62E-08
13	18,770,980	19,046,020	*rs43010890*	Recessive	5.38E-12
13	19,506,748	19,783,272	*rs137476701*	Dominant	1.78E-09
13	20,077,191	20,354,799	*rs133469415*	Additive<br>Dominant	6.14E-14<br>6.14E-14
13	29,397,730	29,684,250	*rs110622330*	Recessive	5.96E-11
13	29,776,357	30,062,520	*rs461403756*	Dominant	1.50E-13
13	38,719,394	39,028,678	*rs110048014*	Additive<br>Recessive	1.37E-08<br>2.94E-10
13	38,961,497	39,272,508	*rs43432233*	Additive<br>Dominant	1.44E-08<br>2.06E-10

13	53,570,673	54,043,428	*rs133341033*	Additive<br>Dominant	2.30E-25<br>2.30E-25
13	65,751,601	66,409,398	*rs137078749*	Additive<br>Dominant	1.38E-08<br>1.05E-08
14	5,977,676	7,007,670	*rs137643258*	Additive<br>Dominant	2.21E-12<br>2.21E-12
14	10,503,090	11,595,996	*rs110963142*	Additive<br>Dominant	1.79E-11<br>3.21E-08
14	10,920,697	12,011,754	*rs109257200*	Recessive	1.03E-08
14	17,427,116	19,026,221	*rs137303686*	Additive<br>Dominant	2.22E-22<br>2.22E-22
14	18,898,727	20,508,616	*rs136484948*	Dominant	3.71E-09
14	21,829,591	23,487,120	*rs132681578*	Dominant	3.64E-09
14	40,373,996	42,513,237	*rs134327452*	Additive<br>Dominant	1.39E-10<br>1.39E-10
14	47,383,242	49,543,299	*rs132969548*	Additive<br>Dominant	5.97E-17<br>5.97E-17
14	48,129,989	50,291,072	*rs41913814*	Recessive	1.90E-13
14	61,186,844	63,368,132	*rs135824064*	Additive<br>Dominant	3.88E-13<br>3.88E-13
14	62,903,956	65,091,197	*rs108988798*	Dominant	2.31E-08
14	63,272,692	65,461,577	*rs136877882*	Additive<br>Dominant	1.48E-09<br>1.48E-09
14	68,298,233	70,639,444	*rs109864366*	Dominant	7.27E-10
14	69,468,403	71,800,181	*rs110724532*	Dominant	4.86E-08
15	3,892,610	4,122,586	*rs43092354*	Dominant	1.92E-08
15	50,236,587	20,566,255	*rs133898251*	Dominant	3.64E-09
15	26,169,835	26,550,309	*rs135503279*	Additive<br>Dominant	6.51E-09<br>2.55E-12
15	27,686,834	28,115,478	*rs109754402*	Dominant	2.64E-08
15	41,454,122	42,017,199	*rs110688968*	Recessive	2.56E-08
15	47,085,829	47,683,465	*rs42709871*	Recessive	4.16E-08
15	49,145,364	49,957,957	*rs42582930**	Recessive	5.77E-09
15	51,694,817	52,472,254	*rs109018952*	Recessive	1.69E-08
15	60,599,380	61,384,207	*rs137823813*	Additive<br>Dominant	2.07E-13<br>2.07E-13
15	67,918,952	68,743,862	*rs42409285*	Additive<br>Dominant	1.07E-14<br>1.07E-14
16	591,660	387,363	*rs133778157*	Additive<br>Dominant	2.99E-33<br>2.99E-33
16	2,652,952	2,481,284	*rs136166247*	Additive<br>Dominant	1.50E-08<br>1.50E-08
16	9,739,230	10,340,087	*rs137501137**	Additive<br>Recessive	1.60E-10<br>5.27E-14
16	28,569,825	29,273,187	*rs133320887*	Dominant	9.97E-09

16	30,188,773	30,938,347	*rs42397367**	Recessive	1.18E-09
16	47,786,357	48,632,055	*rs108984875*	Dominant	3.89E-09
16	52,005,041	53,254,900	*rs137785107*	Additive<br>Dominant	1.19E-24<br>1.19E-24
16	54,652,215	56,109,427	*rs136639933*	Dominant	2.60E-09
16	64,778,352	66,254,121	*rs136982487*	Dominant	1.33E-09
17	26,036	3,910	*rs132751093*	Recessive	1.49E-09
17	517,824	489,394	*rs41834034*	Recessive	2.41E-14
17	3,864,048	3,859,156	*rs41835767*	Dominant	1.91E-08
17	4,547,071	4,548,256	*rs110253313*	Dominant	1.66E-08
17	13,969,248	14,147,211	*rs135563413**	Dominant	8.79E-10
17	39,507,917	40,358,711	*rs110041332*	Dominant	1.59E-08
17	41,252,369	42,216,582	*rs137522961*	Additive<br>Dominant	9.85E-09<br>9.85E-09
17	52,991,529	55,237,847	*rs136539859*	Additive<br>Dominant	1.49E-09<br>1.49E-09
17	55,656,970	57,905,713	*rs41846781*	Dominant	5.31E-12
17	61,351,505	63,626,189	*rs29022475*	Recessive	9.42E-10
17	72,726,732	74,741,425	*rs135830763**	Recessive	1.72E-08
17	72,930,491	74,944,074	*rs133921184**	Additive<br>Dominant	3.35E-37<br>2.93E-37
17	73,036,168	75,073,522	*rs134240868*	Recessive	1.43E-09
18	8,895,233	8,946,233	*rs109049561*	Dominant	3.96E-09
18	21,851,964	21,929,721	*rs41872094*	Additive<br>Dominant	1.22E-08<br>3.85E-10
18	26,260,207	26,345,070	*rs132695972*	Recessive	2.32E-09
18	30,698,212	30,802,833	*rs136230873*	Additive<br>Dominant	4.77E-10<br>4.77E-10
18	51,761,768	52,098,021	*rs135795435*	Additive<br>Dominant	1.29E-08<br>1.51E-12
18	60,746,221	61,024,520	*rs110912084**	Recessive	2.27E-09
18	64,774,970	64,948,024	*rs132853891**	Additive<br>Dominant	2.66E-08<br>2.66E-08
19	7,351,312	7,561,956	*rs109543948*	Dominant	1.49E-09
19	8,798,124	9,030,243	*rs55617305*	Additive<br>Dominant	1.46E-11<br>4.79E-16
19	23,526,814	24,125,771	*rs137270020*	Additive<br>Dominant	1.29E-12<br>1.29E-12
19	52,260,661	52,878,628	*rs42536038**	Recessive	1.62E-08
19	59,314,995	59,887,086	*rs136759305*	Recessive	3.92E-10
20	2,092,588	1,993,220	*rs136404429*	Additive<br>Dominant	5.97E-11<br>5.97E-11
20	12,227,013	12,157,403	*rs42861517*	Dominant	2.53E-10
20	23,863,171	23,886,196	*rs41663374*	Additive<br>Recessive	1.75E-08<br>9.41E-17

20	27,079,454	27,092,889	*rs109295451**	Additive<br>Dominant	3.64E-10<br>1.51E-11
20	32,844,020	32,857,681	*rs41570034*	Additive<br>Dominant	4.92E-08<br>7.25E-09
20	43,870,356	43,878,009	*rs133025074*	Dominant	1.63E-09
20	56,858,708	56,934,353	*rs135510586*	Recessive	5.60E-10
20	67,167,923	67,303,443	*rs41977686*	Dominant	1.65E-08
21	19,865,163	20,266,091	*rs135570367**	Recessive	3.47E-08
22	8,387,721	8,458,003	*rs135197396*	Additive<br>Dominant	1.42E-27<br>1.42E-27
22	14,276,238	14,317,937	*rs41999540*	Dominant	3.55E-08
22	16,592,558	16,637,720	*rs110036873*	Recessive	6.06E-09
22	27,624,256	27,741,860	*rs110812060*	Recessive	1.48E-08
22	28,163,493	28,283,571	*rs110283240*	Recessive	2.29E-12
22	28,460,191	28,580,713	*rs110399563*	Additive<br>Dominant	4.99E-17<br>4.99E-17
22	28,727,485	28,846,011	*rs136708885*	Additive<br>Dominant	3.55E-09<br>3.94E-15
22	35,257,886	35,372,657	*rs109203878*	Additive<br>Dominant	2.00E-15<br>2.82E-16
22	38,471,613	38,612,397	*rs109985339*	Dominant	5.72E-09
22	52,754,203	53,319,295	*rs135350436**	Additive<br>Dominant	4.47E-08<br>4.47E-08
22	54,996,329	55,634,857	*rs109725058*	Recessive	2.62E-13
23	4,277,670	4,197,354	*rs110037746**	Recessive	1.04E-12
23	29,137,447	28,938,184	*rs41622011*	Additive<br>Recessive	4.99E-11<br>2.17E-13
23	29,706,398	29,507,533	*rs110828627*	Dominant	1.48E-11
23	30,716,573	30,467,678	*rs109556465**	Additive<br>Recessive	3.65E-08<br>8.96E-10
23	35,342,022	35,114,464	*rs110684599*	Dominant	2.60E-09
24	5,760,130	6,039,397	*rs43146766**	Dominant	1.75E-08
24	24,898,563	25,207,516	*rs137162280*	Dominant	4.10E-08
24	26,662,068	26,978,274	*rs134272624*	Recessive	1.89E-09
24	39,687,343	40,118,364	*rs110436513*	Dominant	1.50E-08
25	19,679,050	19,891,605	*rs42057006*	Recessive	2.41E-08
26	1,361,088	1,361,105	*rs132818386*	Additive<br>Dominant	2.03E-08<br>2.03E-08
26	7,685,740	7,709,388	*rs133146678*	Dominant	1.11E-13
26	14,468,328	14,512,486	*rs109678718*	Dominant	2.50E-12
26	19,200,857	19,065,913	*rs137211719*	Additive<br>Dominant	4.86E-08<br>6.67E-09
26	28,538,139	28,800,534	*rs42434955*	Additive<br>Dominant	6.95E-09<br>1.99E-09
26	31,462,025	31,743,621	*rs110951772*	Additive	8.62E-10

				Dominant	2.76E-10
26	31,582,567	31,864,154	*rs133291115*	Additive<br>Dominant	7.03E-09<br>7.03E-09
26	32,979,983	33,257,060	*rs42095933**	Additive<br>Dominant	4.03E-08<br>3.52E-13
26	40,532,093	40,905,403	*rs42455375*	Dominant	2.48E-08
27	19,704,072	18,769,799	*rs134936745*	Additive<br>Dominant	8.46E-09<br>4.09E-09
27	19,979,775	19,045,703	*rs42116995*	Recessive	2.18E-12
27	22,017,244	21,083,026	*rs110750240*	Dominant	5.43E-11
27	22,307,598	21,375,791	*rs132728892*	Additive<br>Dominant	5.35E-12<br>5.35E-12
27	35,301,572	34,962,799	*rs135769021**	Additive<br>Dominant	2.68E-10<br>2.68E-10
27	38,840,767	38,687,713	*rs137177737*	Dominant	1.06E-09
28	27,627,548	27,785,987	*rs109367560*	Dominant	1.47E-09
28	29,427,232	29,592,323	*rs136467650*	Additive<br>Dominant	4.14E-08<br>4.14E-08
29	7,809,273	7,868,143	*rs109712075*	Additive<br>Dominant	2.29E-13<br>4.11E-24
29	13,416,477	13,492,098	*rs110880437*	Dominant	7.73E-10
29	19,113,506	19,324,457	*rs42169076**	Additive	9.25E-10
29	25,693,112	25,942,470	*rs136096547*	Additive<br>Dominant	2.24E-08<br>2.24E-08
29	27,237,483	27,614,787	*rs42784046**	Dominant	4.63E-08
29	27,454,921	27,820,784	*rs43170197**	Additive<br>Dominant	9.54E-10<br>8.45E-13
29	29,747,595	30,121,059	*rs109392783*	Dominant	1.75E-14
29	37,919,393	38,495,464	*rs134114922**	Dominant	9.22E-10
29	39,677,851	40,370,794	*rs42189563**	Additive<br>Dominant	8.92E-10<br>3.99E-13
X	7,511,700	7,123,971	*rs109930145*	Dominant	2.29E-10
X	15,770,097	15,668,542	*rs132900305**	Additive<br>Dominant	9.85E-10<br>1.07E-10
X	16,809,410	16,766,595	*rs134765721*	Dominant	1.68E-09
X	52,683,020	58,003,830	*rs137585994*	Additive<br>Dominant	1.67E-17<br>1.67E-17
X	76,226,723	81,257,899	*rs135276595*	Recessive	2.38E-12
X	85,254,844	90,518,686	*rs134541035**	Dominant	6.73E-09
X	132,155,696	139,778,460	*rs137830144*	Additive<br>Dominant	8.09E-09<br>8.09E-09

[1]Chromosome location of the locus; UK refers to an unassigned or unknown chromosomal location.
[2]Single nucleotide polymorphism (SNP) location as measured by numbered nucleotides in reference to the ARS 1.2 genome assembly

(https://www.animalgenome.org/repository/cattle/UMC_bovine_coordinates/; accessed 19 September 2018).

[3]SNP location as measured by numbered nucleotides in reference to the UMD 3.1 genome assembly (http://bovinegenome.org/?q=node/61; accessed 2 April 2018); UK refers to an unassigned or unknown chromosomal location. If the SNP changed chromosomes between build this is noted before the bp position.

[4]The most significant SNP in the locus associated with cow conception rate as identified by *rs* number which is a reference number assigned to markers submitted to the National Center for Biotechnology Information SNP database (https://www.ncbi.nlm.nih.gov/projects/SNP/; accessed 2 April 2018).

[5]SNP located within previously identified copy number variations (CNVs) are denoted with an *.

[6]Genome-wide association model.

[7]Significance (*P*-value) of the most significant SNP associated with cow conception rate.

Table S2. Loci associated with conception rate at first breeding containing transcription factor binding sites.

SNP ID[1]	BTA: position[2]	Transcription Factor(s)[3]	Species[4]	Binding Site[5]	RE Query[6]
rs132895573	1: 36,337	DBP	rat	ATGT[**G**/A]CT	0.0076
rs136034629	1: 16,670,513	Oct-B1	human	ATGCAAA[C/**T**]	0.00124
		POU2F2B	human, rat	ATGCAAA[C/**T**]	0.00124
rs137394358	1: 73,273,238	NF-1/L	rat	TGGC[C/**A**]	0.0039
rs137156811	1: 99,879,710	C/EBPα	human	GAG[T/**C**]AA	0.00834
rs42215584	1: 113,931,474	NF-1/L	rat	T[A/**G**]GCA	0.00976
rs135238817	1: 128,427,500	PR B	human	CACT[**G**/T]TT	0.00686
		PR A	human	CACT[**G**/T]TT	0.00686
		IRF-2	human	TCACT[G/**T**]	0.01609
rs43278206	1: 139,950,787	HOXD9	human, mouse	AAATT[G/**T**]TATT	0.02278
		HOXD10	human, mouse	AAATT[G/**T**]TATT	0.02278
rs108949806	2: 4,883,261	NF-1/L	rat	T[A/**G**]GCA	0.02631
rs137280033	2: 13,518,945	NF-1/L	rat	[**T**/C]GGCA	0.02058
rs110717447	2: 33,688,017	STAT4	human	[**G**/A]GAAAT	0.01064
rs42534341	2: 53,552,010	c-Ets-2	mouse	AGGAAT[G/**T**]C	0.00018
		STAT4	human	GGAAA[G/**T**]	0.00454
		NF-1/L	rat	[G/**T**]GCCA	0.02577
		PEA3	mouse	GGAAA[**G**/T]GC	0.00345
rs41636509	2: 123,768,735	Nrf2:MafK	rat	TCAGAA[**T**/C]	0.00659
		POU2F1	rat	AA[**T**/C]AAG	0.02995
rs136809757	2: 129,876,162	C/EBPα	human	T[**T**/C]GCTC	0.00435
rs133063046	3: 16,653,795	NF-1/L	rat	TG[**G**/A]CA	0.02808
rs136994488	4: 4,700,972	DBP	rat	AG[**C**/A]AAAT	0.01189
rs137111726	4: 9,772,807	NF-1/L	rat	TG[**G**/T]CA	0.02763
rs136800119	4: 48,718,180	c-Ets-2	human	TT[T/**C**]CTC	0.01082
rs132637832	5: 17,922,992	SXR:RXR-α	human	T[**C**/T]TGAACTT	0.00061
		Nrf2:MafK	rat	TTT[**C**/T]TGA	0.01379
rs137251811	5: 21,357,397	HNF-3α	rat	TGTTT[**G**/T]TT	0.04189
		HNF-3β	rat	TGTTT[**G**/T]TT	0.04189
rs135989081	5: 28,218,085	IRF-2	human	TCACT[**T**/G]	0.01048
		PR B	human	CACT[T/**G**]TT	0.00759
		PR A	human	CACT[T/**G**]TT	0.00759
rs134199916	5: 28,939,485	NF-AT1	mouse	ATTTTC[**C**/T]	0.00429
		AR	human	TC[C/**T**]GTTC	0.00204
rs133051543	5: 121,044,113	c-Ets-2	mouse	TC[**A**/C]TTTCCT	0.00071
		POU2F1	rat	CTC[**A**/C]TT	0.01899
		PEA3	mouse	TC[A/**C**]TTTCC	0.02111
		STAT4	human	[**A**/C]TTTCC	0.00873

rs43456078	6: 18,946,287	Nrf2:MafK	rat	TCAGC[C/**A**]T	0.00451
rs135006577	6: 31,792,754	Nrf2:MafK	rat	TTG[**C**/T]TGA	0.01135
rs109934822	6: 80,854,287	STAT4	human	[**G**/A]GAAAT	0.01355
rs134284115	6: 102,407,680	T3R-α	rat	TT[T/**G**]GGGTC	0.00073
rs133675138	7: 81,737,860	Nkx2-1	rat	CTCAA[**G**/A]	0.00432
rs109551298	8: 20,685,523	JunD	mouse	CC[**T**/G]GAC	0.01236
rs109254920	8: 50,283,891	HNF-3α	rat	A[C/**A**]CAAACA	0.00221
rs133697170	8: 66,316,372	RXR-α	human	[A/**G**]GTTCA	0.00576
rs110310103	9: 44,351,018	2-Dec	mouse	GCT[**T**/G]CATTTG	0.00007
rs135920187	9: 98,925,099	C/EBPβ	mouse	AAT[A/**G**]T	0.0335
rs109533748	10: 43,145,489	PR B	human	AACA[A/**G**]TA	0.00706
		PR A	human	AACA[A/**G**]TA	0.00706
rs134880886	10: 92,349,953	NF-AT1	mouse	GGAA[G/**A**]AT	0.00704
		c-Ets-1	human	CAGGAA[**G**/A]	0.00497
rs43661407	11: 13,625,673	NF-1/L	rat	TGGC[**A**/G]	0.02911
rs109265815	11: 16,878,438	Fra-1	rat	[T/**G**]CGTCA	0.02218
rs109465638	11: 22,794,712	HNF-3β	mouse	[A/**C**]AAAACA	0.01359
rs134197588	11: 48,355,243	YY1	mouse	A[**T**/G]GGAG	0.00602
rs110844122	11: 65,350,346	PU.1	mouse	GATGAGG[**A**/C]A	0.00131
		c-Ets-2	human	GAGG[**A**/C]A	0.0134
rs134695420	11: 72,447,958	C/EBPα	human	[A/**G**]AGCAA	0.00734
rs136006377	11: 105,879,823	p53	human	C[C/**A**]TGCCC	0.00315
rs42273278	12: 3,902,971	DBP	rat	AGCA[**A**/G]AC	0.01057
rs134036785	12: 11,261,415	TBP	human	TATAT[G/**A**]AA	0.01541
rs134779941	12: 22,868,176	YY1	human	AAATG[**G**/A]	0.01085
		NF-1/L	rat	TG[**G**/A]CA	0.01415
rs42401835	12: 63,506,888	NF-1/L	rat	TGC[**C**/T]A	0.01517
rs133844853	12: 83,789,534	p53	mouse	CTT[**G**/T]TCTG	0.00282
rs136060818	12: 86,947,480	NF-X3	human	CAGA[**T**/G]GAC	0.001
		XBP-1	human	A[**T**/G]GACT	0.01129
rs110048014	13: 39,028,678	Pax-5	human	GGGCCT[**G**/T]	0.0364
rs136484948	14: 20,508,616	HNF-3β	mouse	TGTTTT[**G**/T]	0.00589
rs42336979<br>*rs41741687*	14: 60,164,356<br>14: 63,273,792	Sp1<br>AP-2αA	pig<br>human	C[**C**/T]GCCC<br>[T/**C**]GGGG	0.02824<br>0.00718
rs43092354	15: 4,122,586	Pax-5	human	CCTGCC[T/**C**]	0.01994
rs133898251	15: 20,566,255	RXR-α	human	TGA[C/**A**]CC	0.00402
rs135503279	15: 26,550,309	HNF-3β	mouse	[**C**/A]AAAACA	0.01181
		C/EBPα	human	GAG[**C**/A]AA	0.03462
rs42709871	15: 47,683,465	Elk-1	human	GAGGAA[**G**/T]	0.04061
rs137823813	15: 61,384,207	POU1F1b	rat	AAAAT[C/**T**]AT	0.02491
		POU1F1c	rat	AAAAT[C/**T**]AT	0.02491
rs136166247	16: 2,481,284	c-Ets-2	human	TTC[T/**C**]TC	0.05109
rs137501137	16: 10,340,087	NF-1	mouse	ATTG[A/**G**]C	0.00565
		Cutl1	mouse	ATTG[A/**G**]C	0.0125

rs42397367	16: 30,938,347	Elk-1	human	CTTC[**C**/T]GC	0.0125
rs136982487	16: 66,254,121	Sp1	pig	[T/**G**]GGCGG	0.04067
rs41835767	17: 3,859,156	NF-1/L	rat	TGG[T/**C**]A	0.02558
rs137522961	17: 42,216,582	NF-AT1	mouse	ATTTT[T/**C**]C	0.002
		TFIID	human	TTT[**T**/C]CAA	0.02093
rs29022475	17: 63,626,189	Fra-1	rat	[**T**/G]GACAC	0.01452
rs132853891	18: 64,948,024	STAT4	human	ATTTC[T/**C**]	0.01793
rs109543948	19: 7,561,956	HNF-3β	mouse	T[T/**G**]TTTTG	0.01099
rs137270020	19: 24,125,771	T3R-β	rat	[**G**/A]GTCACT	0.00102
rs42536038	19: 52,878,628	Pax-5	mouse	[T/**G**]CCACTCCTG	0.00017
		HNF-3β	mouse	TGTTTT[T/**G**]	0.00459
		NF-1/L	rat	T[T/**G**]CCA	0.02674
rs136759305	19: 59,887,086	NF-1/L	rat	T[**G**/T]CCA	0.02388
rs41570034	20: 32,857,681	STAT4	human	[**G**/T]GAAAT	0.01135
	21: 20,266,091	NF-1	mouse	A[**T**/G]TGGC	0.00634
		STAT4	human	GGAAA[**T**/G]	0.00874
		Cutl1	mouse	A[**T**/G]TGGC	0.01684
rs135197396	22: 8,458,003	JunD	mouse	GTCAA[**T**/G]	0.01308
rs110036873	22: 16,637,720	f(alpha)-f(epsilon)	mouse	T[**C**/T]CGGC	0.00646
rs109203878	22: 35,372,657	JunD	mouse	[**T**/G]TCATG	0.00817
rs135350436	22: 53,319,295	HNF-1C	human	AAAT[**T**/C]GC	0.00402
rs41622011	23: 28,938,184	T3R-β	rat	GGTCA[**C**/T]C	0.00209
rs133537811	24: 6,067,867	NF-1/L	rat	TGG[**C**/A]A	0.02058
rs110436513	24: 40,118,364	HNF-3β	mouse	TGTTTT[**G**/T]	0.00743
rs133291115	26: 31,864,154	TFIID	human	TTTTC[**T**/CA]	0.04343
rs110880437	29: 13,492,098	HNF-1A	human	TAATT[G/**A**]T	0.04374
		HNF-1B	human	TAATT[G/**A**]T	0.04374
		HNF-1C	human	[**G**/A]TAATTT	0.01426
rs136096547	29: 25,942,470	Pax-5	human	G[**G**/A]GCTTG	0.03234
rs134114922	29: 38,495,464	LF-A1	human	CAG[**G**/T]GCA	0.00302
rs42916622	29: 40,348,542	NF-1/L	rat	[G/**T**]GGCA	0.01871
rs137585994	X: 58,003,830	ARP-1	rat	T[**A**/G]ACCTTTG	0.00042
		JunD	mouse	CTT[**A**/G]AC	0.01397
rs137830144	X: 139,778,460	myogenin	mouse	CCT[**G**/A]CTGC	0.00186
		p53	mouse	CTTGCCT[**G**/A]	0.00469

[1]The most significant SNP in the locus as identified by *rs* number which is a reference number assigned to markers submitted to the National Center for Biotechnology Information SNP database (https://www.ncbi.nlm.nih.gov/projects/SNP/; accessed 2 April 2018).
[2]Single nucleotide polymorphism (SNP) location as measured by numbered nucleotides in reference to the UMD 3.1 genome assembly (http://bovinegenome.org/?q=node/61; accessed 2 April 2018).
[3]Gene symbol for the transcription factors whose binding sites spanned the locus.
[4]Speices that the transcription factor binding site (TFBS) was identified.

[5]Binding site for the transcription factor, with the SNP mutation in brackets. Bolded alleles are associated with the TFBS.

[6]Random expectation (RE) query values as calculated by the PROMO (http://alggen.lsi.upc.es/cgi-bin/promo_v3/promo/promoinit.cgi?dirDB=TF_8.3; accessed 25 July 2018).

Table S3. Loci associated with number of times bred to conception.

BTA[1]	ARS 1.2 BP Position[2]	UMD 3.1 Position[3]	SNP ID[4,5]	Model[6]	P-Value[7]
UK	UK	BTAX 20,919,326	*rs134105181*	Dominant	4.07E-08
1	33,136,647	32,700,496	*rs43226666**	Dominant	9.31E-11
1	61,470,011	62,056,267	*rs42760220*	Dominant	2.54E-08
1	82,457,094	83,047,838	*rs109289667*	Recessive	2.36E-08
1	99,081,740	99,879,710	*rs137156811*	Additive<br>Dominant	2.39E-18<br>2.39E-18
1	113,023,021	113,931,474	*rs42215584*	Additive<br>Dominant	6.74E-16<br>7.67E-17
1	127,938,641	128,938,068	*rs43265135**	Additive<br>Dominant	4.42E-10<br>1.14E-11
2	4,950,986	4,883,261	*rs108949806*	Dominant	3.63E-09
2	86,075,750	86,479,252	*rs133738177*	Additive<br>Dominant	1.01E-15<br>1.01E-15
2	123,224,834	123,768,735	*rs41636509**	Recessive	2.01E-08
3	23,385,126	23,494,800	*rs134056476*	Additive<br>Dominant	2.10E-11<br>7.15E-12
3	26,320,263	26,436,103	*rs109659431*	Additive	7.52E-09
3	31,645,740	31,800,315	*rs43746101*	Recessive	1.93E-08
3	54,398,276	54,575,757	*rs110480314**	Additive<br>Dominant	1.54E-08<br>2.92E-11
3	72,002,117	72,296,340	*rs29013285*	Dominant	2.49E-08
3	80,914,344	81,245,225	*rs43349445*	Dominant	2.97E-08
3	97,093,984	97,680,312	*rs41585055*	Additive<br>Dominant	1.02E-08<br>1.55E-12
3	97,504,864	98,096,750	*rs29024665*	Dominant	2.78E-09
3	97,690,487	98,284,456	*rs43360769*	Recessive	4.42E-08
3	110,948,044	111,540,905	*rs133945887*	Recessive	7.25E-12
4	37,056,340	37,235,676	*rs133417267*	Additive<br>Dominant	4.49E-10<br>4.49E-10
4	43,184,336	43,419,900	*rs109009636*	Recessive	1.80E-08
4	92,464,322	93,277,289	*rs109721084*	Additive<br>Dominant	4.28E-10<br>1.33E-10
5	4,361,199	4,346,709	*rs110152722**	Additive<br>Dominant	6.70E-09<br>4.07E-09
5	12,616,464	12,688,864	*rs136793667*	Additive	1.01E-08
5	17,857,463	17,922,992	*rs132637832*	Additive<br>Dominant	2.73E-09<br>2.73E-09
5	28,774,907	28,939,485	*rs134199916**	Additive<br>Dominant	1.81E-08<br>1.81E-08

5	30,438,787	30,608,814	*rs133335343**	Additive<br>Dominant	1.39E-09<br>1.09E-10
5	36,663,436	36,860,611	*rs136252597*	Additive<br>Dominant	1.41E-10<br>1.41E-10
5	59,553,709	59,905,836	*rs137261569**	Additive<br>Dominant	7.75E-26<br>7.75E-26
5	110,537,227	111,051,266	*rs136545177**	Recessive	5.50E-09
6	7,448,715	8,628,487	*rs136663821*	Dominant	5.59E-10
6	10,139,671	11,301,952	*rs29019244*	Dominant	5.86E-09
6	10,478,297	11,640,946	*rs108963104**	Dominant	1.16E-08
6	10,734,724	11,898,659	*rs137187491*	Additive<br>Dominant	3.64E-08<br>3.64E-08
6	17,693,932	18,946,287	*rs43456078*	Recessive	2.32E-09
6	30,369,047	31,792,754	*rs135006577*	Additive<br>Dominant	4.60E-18<br>4.60E-18
6	37,013,919	38,447,022	*rs109381958**	Additive<br>Dominant	4.39E-26<br>4.39E-26
6	53,086,414	54,689,211	*rs42762416*	Additive<br>Dominant	1.49E-11<br>1.49E-11
6	79,147,024	80,854,287	*rs109934822**	Recessive	6.90E-09
6	91,908,296	93,660,111	*rs132854961*	Recessive	8.38E-12
6	98,949,730	100,733,028	*rs43488004*	Additive<br>Dominant	5.59E-10<br>1.68E-12
7	176,899	151,277	*rs43114893*	Recessive	1.61E-08
7	1,149,677	1,053,454	*rs133295796*	Additive<br>Dominant	1.00E-17<br>100E-17
7	7,199,859	8,223,914	*rs133396251**	Additive<br>Dominant	1.15E-09<br>4.78E-11
7	59,565,353	61,564,301	*rs42852759**	Recessive	4.24E-08
7	78,348,022	80,634,324	*rs132636869*	Dominant	9.02E-10
7	97,540,689	99,952,417	*rs110327822*	Dominant	5.99E-10
8	20,675,446	20,685,523	*rs109551298*	Recessive	7.94E-09
8	20,721,025	20,731,035	*rs43539285*	Additive<br>Dominant	7.91E-11<br>1.34E-10
8	29,565,411	29,615,889	*rs134405734*	Additive<br>Dominant	1.25E-24<br>1.25E-24
8	58,921,513	59,331,066	*rs136685338*	Additive<br>Dominant	1.60E-08<br>1.34E-08
8	65,837,664	66,316,372	*rs133697170*	Additive<br>Dominant	4.44E-11<br>4.44E-11
8	77,981,766	79,362,912	*rs134979587*	Additive<br>Dominant	9.72E-11<br>9.72E-11
8	111,550,769	UK	*rs134584163*	Additive<br>Dominant	1.24E-11<br>1.24E-11

9	6,538,240	6,661,699	*rs110968046**	Recessive	3.57E-08
9	43,839,708	44,351,018	*rs110310103*	Additive<br>Dominant	1.26E-08<br>1.66E-10
9	64,893,414	65,812,782	*rs110885320*	Recessive	1.60E-08
9	74,687,743	75,695,000	*rs134083077*	Additive<br>Dominant	3.76E-13<br>3.76E-13
10	43,101,892	43,145,489	*rs109533748*	Additive<br>Dominant	1.37E-08<br>1.37E-08
10	62,992,388	63,217,046	*rs133388647*	Recessive	1.18E-08
10	67,250,158	67,481,157	*rs29026382*	Recessive	6.72E-10
10	76,396,374	76,708,701	*rs132793686*	Dominant	2.30E-10
10	80,522,688	80,873,088	*rs110456535*	Dominant	1.57E-09
11	16,856,558	16,878,438	*rs109265815*	Dominant	1.01E-08
11	77,409,930	77,475,988	*rs109117330*	Recessive	4.24E-08
11	106,236,174	105,879,823	*rs136006377*	Additive<br>Dominant	1.08E-13<br>1.08E-13
12	62,999,694	63,506,888	*rs42401835**	Additive<br>Dominant	7.74E-10<br>7.74E-10
13	18,770,980	19,046,020	*rs43010890*	Recessive	2.73E-09
13	20,077,191	20,354,799	*rs133469415*	Additive<br>Dominant	1.79E-09<br>1.79E-09
13	29,776,357	30,062,520	*rs461403756*	Dominant	1.81E-10
13	53,570,673	54,043,428	*rs133341033*	Additive<br>Dominant	1.03E-20<br>1.36E-20
13	65,751,601	66,409,398	*rs137078749*	Additive<br>Dominant	6.52E-09<br>8.70E-09
14	5,717,831	6,746,602	*rs109419409*	Additive<br>Dominant	7.86E-10<br>2.39E-10
14	17,427,116	19,026,221	*rs137303686*	Additive<br>Dominant	3.02E-14<br>3.02E-14
14	47,383,242	49,543,299	*rs132969548*	Additive<br>Dominant	1.68E-12<br>1.68E-12
14	48,129,989	50,291,072	*rs41913814*	Recessive	3.35E-09
14	61,186,844	63,368,132	*rs135824064*	Additive<br>Dominant	2.15E-09<br>2.15E-09
15	26,169,835	26,550,309	*rs135503279*	Dominant	1.09E-09
15	27,686,834	28,115,478	*rs109754402*	Dominant	1.08E-08
15	49,145,364	49,957,957	*rs42582930**	Recessive	3.08E-08
15	60,599,380	61,384,207	*rs137823813*	Additive<br>Dominant	2.28E-09<br>2.28E-09
15	67,918,952	68,743,862	*rs42409285*	Additive<br>Dominant	3.01E-08<br>3.01E-08
16	591,660	387,363	*rs133778157*	Additive<br>Dominant	5.68E-28<br>5.68E-28

16	9,739,230	10,340,087	*rs137501137**	Additive<br>Recessive	5.11E-10<br>1.12E-13
16	30,188,773	30,938,347	*rs42397367**	Recessive	1.13E-08
16	52,005,041	53,254,900	*rs137785107*	Additive<br>Dominant	4.24E-16<br>4.24E-16
17	517,824	489,394	*rs41834034*	Recessive	4.57E-09
17	13,969,248	14,147,211	*rs135563413**	Dominant	2.08E-08
17	31,892,333	32,284,932	*rs110556114*	Dominant	4.90E-08
17	55,656,970	57,905,713	*rs41846781*	Dominant	1.85E-10
17	69,370,793	71,523,508	*rs133533188*	Additive	9.49E-09
17	72,930,491	74,944,074	*rs133921184**	Additive<br>Dominant	2.59E-23<br>5.98E-22
17	73,036,168	75,073,522	*rs134240868*	Recessive	2.08E-08
18	8,418,469	8,467,054	*rs133533188*	Additive<br>Dominant	4.34E-08<br>4.20E-08
18	8,895,233	8,946,233	*rs109049561*	Dominant	1.11E-08
18	26,260,207	26,345,070	*rs132695972*	Recessive	4.36E-08
18	49,932,292	50,210,007	*rs109816901*	Additive	3.21E-08
18	51,761,768	52,098,021	*rs135795435*	Additive<br>Dominant	2.24E-08<br>7.63E-12
19	2,455,040	2,536,668	*rs109414660**	Dominant	6.43E-09
19	8,798,124	9,030,243	*rs55617305*	Additive<br>Dominant	1.20E-08<br>1.59E-13
20	2,092,588	1,993,220	*rs136404429*	Additive<br>Dominant	4.90E-08<br>4.90E-08
20	23,863,171	23,886,196	*rs41663374*	Recessive	1.58E-11
20	27,079,454	27,092,889	*rs109295451**	Dominant	3.28E-08
20	56,858,708	56,934,353	*rs135510586*	Recessive	1.36E-08
21	24,108,341	24,575,987	*rs137835919*	Additive	4.93E-08
22	8,387,721	8,458,003	*rs135197396*	Additive<br>Dominant	3.37E-19<br>3.37E-19
22	28,163,493	28,283,571	*rs110283240*	Recessive	1.18E-08
22	28,460,191	28,580,713	*rs110399563*	Additive<br>Dominant	4.38E-13<br>4.38E-13
22	28,727,485	28,846,011	*rs136708885*	Dominant	2.69E-12
22	35,257,886	35,372,657	*rs109203878*	Additive<br>Dominant	1.61E-12<br>1.27E-13
22	38,471,613	38,612,397	*rs109985339*	Dominant	1.76E-08
22	54,996,329	55,634,857	*rs109725058*	Recessive	2.92E-13
23	4,277,670	4,197,354	*rs110037746**	Recessive	6.23E-11
23	29,137,447	28,938,184	*rs41622011*	Additive<br>Recessive	9.36E-09<br>3.47E-08
23	29,706,398	29,507,533	*rs110828627*	Dominant	4.53E-09

23	35,342,022	35,114,464	*rs110684599*	Dominant	2.95E-09
26	7,685,740	7,709,388	*rs133146678*	Dominant	1.65E-11
26	14,468,328	14,512,486	*rs109678718*	Dominant	1.83E-09
26	31,462,025	31,743,621	*rs110951772*	Additive<br>Dominant	2.65E-08<br>1.84E-08
26	32,979,983	33,257,060	*rs42095933**	Dominant	1.13E-12
27	22,017,244	21,083,026	*rs110750240*	Dominant	4.93E-08
27	22,307,598	21,375,791	*rs132728892*	Additive<br>Dominant	2.96E-08<br>2.96E-08
27	38,840,767	38,687,713	*rs137177737*	Dominant	9.90E-09
28	27,627,548	27,785,987	*rs109367560*	Dominant	3.21E-09
29	7,809,273	7,868,143	*rs109712075*	Additive<br>Dominant	5.21E-12<br>1.17E-19
29	19,113,506	19,324,457	*rs42169076**	Additive	3.76E-08
29	27,454,921	27,820,784	*rs43170197**	Additive<br>Dominant	2.67E-08<br>1.21E-10
29	29,747,595	30,121,059	*rs109392783*	Dominant	2.06E-13
29	39,677,851	40,370,794	*rs42189563**	Additive<br>Dominant	1.70E-08<br>5.49E-11
29	49,668,181	50,307,491	*rs41586957**	Recessive	2.83E-08
X	7,511,700	7,123,971	*rs109930145*	Dominant	2.56E-11
X	15,770,097	15,668,542	*rs132900305**	Dominant	3.37E-09
X	52,683,020	58,003,830	*rs137585994*	Additive<br>Dominant	1.44E-13<br>1.44E-13

[1]Chromosome location of the locus; UK refers to an unassigned or unknown chromosomal location.
[2]Single nucleotide polymorphism (SNP) location as measured by numbered nucleotides in reference to the ARS 1.2 genome assembly (https://www.animalgenome.org/repository/cattle/UMC_bovine_coordinates/; accessed 19 September 2018).
[3]SNP location as measured by numbered nucleotides in reference to the UMD 3.1 genome assembly (http://bovinegenome.org/?q=node/61; accessed 2 April 2018); UK refers to an unassigned or unknown chromosomal location. If the SNP changed chromosomes between build this is noted before the bp position.
[4]The most significant SNP in the locus associated with cow conception rate as identified by *rs* number which is a reference number assigned to markers submitted to the National Center for Biotechnology Information SNP database (https://www.ncbi.nlm.nih.gov/projects/SNP/; accessed 2 April 2018).
[5]SNP located within previously identified copy number variations (CNVs) are denoted with an *****.
[6]Genome-wide association model.
[7]Significance (*P*-value) of the most significant SNP associated with cow conception rate.

Table S4. Loci associated with number of times bred to conception containing transcription factor binding sites.

SNP ID[1]	BTA: position[2]	Transcription Factor(s)[3]	Species[4]	Binding Site[5]	RE Query[6]
rs137156811	1: 99,879,710	C/EBPα	human	GAG[T/**C**]AA	0.00834
rs42215584	1: 113,931,474	NF-1/L	rat	T[A/**G**]GCA	0.00976
rs108949806	2: 4,883,261	NF-1/L	rat	T[A/**G**]GCA	0.02631
rs41636509	2: 123,768,735	Nrf2:MafK	rat	TCAGAA[C/**T**]	0.00659
		POU2F1	rat	AA[C/**T**]AAG	0.02995
rs132637832	5: 17,922,992	SXR:RXR-α	human	T[**C**/T]TGAACTT	0.00061
		Nrf2:MafK	rat	TTT[**C**/T]TGA	0.01379
rs134199916	5: 28,939,485	NF-AT1	mouse	ATTTTC[**C**/T]	0.00429
		AR	human	TC[C/**T**]GTTC	0.00204
rs43456078	6: 18,946,287	Nrf2:MafK	rat	TCAGC[C/**A**]T	0.00451
rs135006577	6: 31,792,754	Nrf2:MafK	rat	TTG[**C**/T]TGA	0.01135
rs109934822	6: 80,854,287	STAT4	human	[**G**/A]GAAAT	0.01355
rs43114893	7: 151,277	GR	mouse	A[A/**G**]AACT	0.00976
rs109551298	8: 20,685,523	JunD	mouse	CC[**T**/G]GAC	0.01236
rs133697170	8: 66,316,372	RXR-α	human	[A/**G**]GTTCA	0.00576
rs110310103	9: 44,351,018	2-Dec	mouse	GCT[**T**/G]CATTTG	0.00007
rs109533748	10: 43,145,489	PR B	human	AACA[A/**G**]TA	0.00706
		PR A	human	AACA[A/**G**]TA	0.00706
rs109265815	11: 16,878,438	Fra-1	rat	[T/**G**]CGTCA	0.02218
rs136006377	11: 105,879,823	p53	human	C[C/**A**]TGCCC	0.00315
rs42401835	12: 63,506,888	NF-1/L	rat	TGC[**C**/T]A	0.01517
rs42336979	14: 60,164,356	Sp1	pig	C[**C**/T]GCCC	0.02824
rs135503279	15: 26,550,309	HNF-3β	mouse	[**C**/A]AAAACA	0.01181
		C/EBPα	human	GAG[**C**/A]AA	0.03462
rs137823813	15: 61,384,207	POU1F1b	rat	AAAAT[C/**T**]AT	0.02491
		POU1F1c	rat	AAAAT[C/**T**]AT	0.02491
rs137501137	16: 10,340,087	NF-1	mouse	ATTG[A/**G**]C	0.00565
		Cutl1	mouse	ATTG[A/**G**]C	0.0125
rs42397367	16: 30,938,347	Elk-1	human	CTTC[**C**/T]GC	0.0125
rs137835919	21: 24,575,987	NF-E2	mouse	ACC[C/**A**]TGACTCAGC	0
		XBP-1	human	[C/**A**]TGACT	0.00606
		JunD	mouse	C[C/**A**]TGAC	0.00903
rs135197396	22: 8,458,003	JunD	mouse	GTCAA[**T**/G]	0.01308
rs109203878	22: 35,372,657	JunD	mouse	[**T**/G]TCATG	0.00817
rs41622011	23: 28,938,184	T3R-β	rat	GGTCA[**C**/T]C	0.00209
rs42916622	29: 40,348,542	NF-1/L	rat	[G/**T**]GGCA	0.01871
rs137585994	X: 58,003,830	ARP-1	rat	T[**A**/G]ACCTTTG	0.00042
		JunD	mouse	CTT[**A**/G]AC	0.01397

[1]The most significant SNP in the locus as identified by *rs* number which is a reference number assigned to markers submitted to the National Center for Biotechnology Information SNP database (https://www.ncbi.nlm.nih.gov/projects/SNP/; accessed 2 April 2018).
[2]Single nucleotide polymorphism (SNP) location as measured by numbered nucleotides in reference to the UMD 3.1 genome assembly (http://bovinegenome.org/?q=node/61; accessed 2 April 2018).
[3]Gene symbol for the transcription factors whose binding sites spanned the locus.
[4]Speices that the transcription factor binding site was identified in.
[5]Binding site for the transcription factor, with the SNP mutation in brackets. Bolded alleles are associated with the TFBS.

[6]Random expectation (RE) query values as calculated by the PROMO (http://alggen.lsi.upc.es/cgi-bin/promo_v3/promo/promoinit.cgi?dirDB=TF_8.3; accessed 25 July 2018).

Table S5. List of positional candidate genes found to be differentially expressed by Moraes et al., (2018).

DE Comparison(s)[1]	Gene	GWAA Phenotype(s)[2]
Pregnant Endometrium: High Fertile vs Subfertile<br>High Fertile: Pregnant vs Open Endometrium<br>Subfertile: Pregnant vs Open Endometrium		
	NTRK2	Both
	OAS2	CCR1
Pregnant Endometrium: High Fertile vs Subfertile<br>High Fertile: Pregnant vs Open Endometrium		
	APH1B	CCR1
	COL6A3	CCR1
	PSD3	Both
	PTGFRN	Both
High Fertile: Pregnant vs Open Endometrium<br>Subfertile: Pregnant vs Open Endometrium		
	APOBEC3A	Both
	ARHGAP15	CCR1
	LOC512869	CCR1
	LOC781710	Both
	MX1	CCR1
	OAS1Y	CCR1
	ROBO1	CCR1
	TMTC2	Both
Pregnant Endometrium: High Fertile vs Subfertile<br>Conceptuses: High Fertile vs Subfertile		
	RGL1	CCR1
High Fertile: Pregnant vs Open Endometrium		
	ADD2	CCR1
	CA12	CCR1
	CDH23	Both
	FAM171A1	Both
	HS6ST2	CCR1
	LHFP	CCR1
	LOC100848714	Both
	MAGI2	Both
	MUM1	CCR1
	NDRG3	Both
	NFASC	CCR1
	NUMBL	TBRD
	OXCT1	CCR1
	PDZRN3	Both
	PRDM1	Both
	SF3A1	TBRD

	SHROOM3	CCR1
	SIK3	Both
	SLC8A1	CCR1
	SPATS2	Both
	SYNE2	Both
	UGCG	CCR1
	ZFP37	CCR1
	ZNF331	CCR1
Conceptuses: High Fertile vs Subfertile		
	ACADL	CCR1
	ENAH	CCR1
	FAHD2A	CCR1
	FZD6	Both
	GJB5	Both
	LOC104970773	CCR1
	LOC504861	CCR1
	LOC521902	Both
	NOD1	CCR1
	NOTCH2	Both
	PRKG1	Both
	RNF224	Both
	SLC6A11	Both
	ST3GAL4	Both

[1]Name of the fertility groups compared by Moraes et al. (2018).
[2]Phenotype(s) the positional candidate gene was associated with in the genome-wide association analysis (GWAA): cow conception rate at first AI (CCR1), number of times a cow was bred to achieve conception (TBRD), or both.
